# Workplace harassment is associated with differences in personality traits, coping strategies and work ability: cross sectional study among healthcare professionals

**DOI:** 10.3389/fpubh.2025.1641654

**Published:** 2025-09-03

**Authors:** Silvia Vivarelli, Caterina Oliveri, Saveria Savasta, Francesca Simona Fiorino, Concettina Fenga

**Affiliations:** Department of Biomedical and Dental Sciences, Morphological and Functional Imaging, Section of Occupational Medicine, University of Messina, Messina, Italy

**Keywords:** workplace harassment (WPH), healthcare workers (HW), psychodiagnostics questionnaires, workplace health promotion (WHP), occupational health

## Abstract

**Introduction:**

Workplace Harassment (WPH) in the healthcare sector remains a critical global issue, adversely affecting employees’ psychological well-being and work ability.

**Aim:**

This study investigated gender differences, variations in personality traits and coping strategies linked to WPH, and the impact of these dynamics on work performance of healthcare workers (HCW).

**Methods:**

A cross-sectional survey was administered to a sample of 415 HCW (138 men and 277 women), including physicians, nurses, and other staff members (e.g., administrative, technical, and auxiliary personnel). Participants completed a series of standardized instruments: WPH and Health Survey Questionnaire (WHHSQ), Mini International Personality Item Pool (Mini-IPIP), Brief-COPE inventory, and Work Ability Index (WAI).

**Results:**

Reports of WPH within the past 12 months showed a significantly higher prevalence among women (36.2%, *n* = 94) compared to men (23.3%, *n* = 31), *p* < 0.01. Verbal abuse (30.4%) and bullying/mobbing (17.1%) were the most common forms, with supervisors implicated in about 10–15% of cases. Informal handling, such as confiding in colleagues or family (20–30%), was far more frequent than formal reporting (8–10%). Females scored higher in Agreeableness (*M* = 16.26 vs. 15.29, *p* < 0.001) and Conscientiousness (*M* = 15.03 vs. 14.01, *p* < 0.001) but lower in Neuroticism (*M* = 11.56 vs. 12.93, *p* < 0.0001) compared to males. Individuals who experienced harassment, exhibited lower Neuroticism (mean = 11.28 vs. 12.37, *p* < 0.0001) than non-harassed peers. Coping patterns varied: women reported greater use of Seeking Social Support strategies (*M* = 17.80 vs. 16.02, *p* < 0.0001), while harassed individuals relied more on Avoidance strategies (*M* = 19.15 vs. 18.16, *p* < 0.05), including self-distraction and substance use. Men had higher work ability scores than women (*χ*^2^ = 8.799, *p* < 0.05), while WPH was linked to a significant reduction in work ability (*χ*^2^ = 15.729, *p* < 0.01).

**Conclusion:**

Women are likely to face higher WPH rates and tend to seek social support more frequently, while harassed individuals, regardless of gender, increasingly rely on avoidance coping. Exposure to WPH is associated with decreased work ability. Low rate of formal reporting reveals systemic gaps in institutional responses. To address these challenges, healthcare organizations should implement comprehensive risk assessment strategies incorporating gender-specific factors and psychological profiles to identify vulnerable staff earlier. Enhancing reporting systems, offering proactive psychological support, and promoting adaptive coping strategies are essential to reduce harm, foster resilience, thereby creating safer and healthier work environments.

## 1 Introduction

Workplace violence represents a significant global public health issue, especially in healthcare organizations (1, 2). The International Labour Organisation (ILO) defines workplace violence as “*any action, incident or behavior that departs from reasonable conduct in which a person is assaulted, threatened, harmed, (or) injured in the course of, or as a direct result of, his or her work*” (3). It can be categorized into two main types: physical and psychological. Physical violence involves the use of force against an individual or group, potentially resulting in physical, sexual, or psychological harm (4). Psychological violence, such as the deliberate exertion of power, includes harassment forms such as verbal abuse, bullying or mobbing, and sexual or racial discrimination (5, 6). While physical violence has long been acknowledged, workplace harassment (WPH) has historically been underestimated and only recently has begun to receive appropriate attention, representing a threat in several working environments (7).

Due to the nature of healthcare settings, which require continuous interpersonal interaction, rapid decision-making, as well as emotional payloads, healthcare workers (HCWs) face elevated risk of workplace violence, with increasing frequency over time (8). Emergency and intensive care units are particularly prone to such episodes. Communication difficulties, emotional distress of patients, and clinician fatigue can exacerbate tensions, increasing the risk for encountering either violence or harassment situations (9). Notably, a surge in violence was observed during the COVID-19 pandemic, with more than half of HCWs reporting exposure to workplace violence episodes (10). Despite widespread attention to pandemic-related burdens, the psychological and organizational consequences of such violence, especially the harassment, remain underappreciated and warrant further investigation (11).

Globally, workplace violence in healthcare is widespread, with Asia and Latin America showing the highest rates of reported incidents. Gender-based disparities are significant: women, particularly in these regions, are disproportionately affected (12, 13). Evidence from other regions, such as New Zealand, also confirms the high prevalence of workplace bullying in healthcare settings, including pharmacy, where underreporting and psychological impacts are common (14). In Italy, between 2020 and 2022, approximately 6,000 cases of workplace violence were reported in the healthcare sector, accounting for 41% of all episodes in the services and industrial sectors during the same period (15). Recent data confirm that workplace violence is particularly prevalent in psychiatric and emergency departments and predominantly affects nurses and physicians (16). In Italy, 12–93% of HCWs reported verbal aggression and threats, while 28–50% reported physical violence (17).

In this context, individual psychological characteristics, such as personality traits and coping strategies, have emerged as critical factors in understanding both vulnerability to and the psychological impact of WPH (18). The relationship between harassment and personality traits have been investigated by a number of studies, although the results are often controversial (19). While some findings demonstrated that certain personality dimensions may influence how individuals perceive, react to, and cope with hostile work environments, other ones show that personality traits are not different between harassed and not harassed (20, 21). Theoretical models of harassment showed a relationship between neuroticism and harassment (22). In details, subjects with high levels of neuroticism are more likely to experience elevated stress responses and interpret ambiguous situations as threatening, making them more susceptible to emotional strain and potentially increasing the likelihood of perceiving harassment (23). Conversely, traits such as conscientiousness and agreeableness have been associated with more constructive interpersonal interactions and may act as protective factors against some of the adverse effects of workplace conflict and aggression (24).

In parallel, coping strategies, defined as cognitive and behavioral efforts to manage psychological stress, play a crucial role in moderating the effects of WPH (25). Adaptive strategies, such as seeking social support, problem-solving, and positive reframing, can help mitigate the psychological burden of harassment and foster resilience (26). In contrast, maladaptive strategies, including denial, disengagement, and substance use, are often linked to worse outcomes, such as heightened anxiety, burnout, and decreased work ability (27, 28).

Gender may further influence both personality expression and coping styles, potentially shaping differential exposure to WPH and its consequences (29). In particular, women have been shown to report higher levels of neuroticism and to employ more emotion-focused coping strategies, such as seeking social support or rumination, which may heighten psychological vulnerability in high-stress environments (22). Men, on the other hand, are more likely to adopt problem-focused or avoidant coping mechanisms, which could either buffer or exacerbate the impact of harassment depending on context (30). These gendered patterns not only affect how harassment is experienced and managed, but may also influence reporting behaviors and perceived legitimacy of psychological distress in workplace settings (31). Despite their importance, the interplay between individual traits, coping behaviors, and WPH remains insufficiently explored in healthcare settings, particularly within the Italian context.

Beyond individual psychological responses, workplace violence has been associated with a wide range of organizational and functional impairments, including reduced work ability, which is a multidimensional construct that incorporates workers’ physical and mental health, professional competence, and the capacity to meet job demands safely and effectively (32). While existing literature has linked reduced work ability to chronic stress, burnout, comorbid health conditions, and demanding shift patterns, the role of WPH as a determinant of work ability remains insufficiently addressed. Investigating this association is critical, given the implications for workforce sustainability and patient care quality (33). Notably, personality traits and coping strategies may act as mediating or moderating factors in the relationship between WPH and work ability, potentially amplifying or mitigating its negative effects (34). Understanding how these psychological and organizational dimensions intersect with harassment exposure may provide a more comprehensive model of risk and resilience within healthcare environments.

Based on this background, the primary aim of this study is to investigate the prevalence and characteristics of psychological harassment in a university hospital in Southern Italy, with particular attention to gender differences. Furthermore, the study aims to examine how personality traits and coping strategies may influence both exposure to harassment and the behavioral response to it. Lastly, this research explores the relationship between WPH and work ability, to better understand the functional consequences of such perpetrations in healthcare settings. The finding may help to develop innovative risk assessment frameworks and support the design of targeted environmental and organizational interventions for the effective prevention of workplace harassment.

## 2 Methods

### 2.1 Study design and population

A self-administered electronic survey was used as for harassment, work and neurobehavioral assessment among healthcare personnel. Data were collected from September 1 2024 to January 31 2025, involving both medical and non-medical staff employed at a University Hospital in Southern Italy. Inclusion criteria were: (1) current employment at the hospital during the survey period, (2) age ≥ 18 years, and (3) provision of informed consent. Survey responses that were incomplete or did not meet any of the inclusion criteria were excluded from further analysis. Survey invitations were disseminated via institutional email, the hospital’s official website, and affiliated social media platforms. Participation was anonymous and voluntary. Informed consent and study details were provided at the beginning of the survey. A total of 437 healthcare workers were approached, and 415 participants (95%) met the inclusion criteria, comprising 138 men (33.3%) and 277 women (66.7%).

### 2.2 Assessment tools

#### 2.2.1 Workplace harassment in health service questionnaire (WHHSQ)

Workplace harassment (WPH) was assessed using a shortened and adapted version of the WHO’s Workplace Violence in Health Services Sector Questionnaire, translated into Italian by the Nursing Up Union (35, 36). This adapted version specifically focuses on psychological forms of violence rather than workplace violence in general. It includes six sections: sociodemographic data, workplace characteristics and experience, verbal aggression, bullying/mobbing, sexual harassment, and racially motivated harassment. The questionnaire collects detailed information on age, gender, job role, years of work experience, work environment, shift patterns, and health status. Each type of harassment is rated on a Likert scale measuring frequency (from “never” to “very often”) and psychological impact. Higher scores reflect greater exposure and psychological burden. Total scores were used to categorize exposure levels and facilitate comparisons across professional roles, departments, and demographic subgroups. Data were also stratified by gender to examine disparities in the prevalence and severity of harassment.

#### 2.2.2 Mini-IPIP questionnaire

Personality traits were measured using the 20-item Mini-IPIP, a validated abbreviated instrument assessing the Big Five personality dimensions, according to Goldberg (37). In detail, these are Openness (creativity and openness to new experiences), Conscientiousness (organization and dependability), Extraversion (sociability and assertiveness), Agreeableness (compassion and cooperativeness), and Neuroticism (emotional instability and tendency toward negative emotions) (38). Each dimension was evaluated through four items rated on a 5-point Likert scale ranging from 1 (Strongly Disagree) to 5 (Strongly Agree), resulting in subscale scores that ranged from 4 to 20. Scores were further categorized into five interpretive levels: Very Low (4–7), Low (8–10), Moderate (11–13), High (14–16), and Very High (17–20), allowing for the classification of individual personality profiles (39). To investigate potential associations between personality and workplace dynamics, data were further stratified by gender and by exposure to workplace harassment (yes/no).

#### 2.2.3 Brief-COPE questionnaire

Coping strategies were measured using the Brief-COPE, a validated 28-item self-report instrument assessing responses to stress across 14 specific strategies: i.e., emotional support, instrumental support, venting, religion, active coping, planning, disengagement, self-blame, self-distraction, denial, substance use, positive reframing, acceptance, and humor. Each item is rated on a 4-point Likert scale (1 = “Not at all” to 4 = “Very often”) (40). For analysis, strategies were grouped into four higher-order dimensions based on theoretical models: Seeking Social Support (8 items, 4 strategies, i.e., emotional support, instrumental support, venting, and religion, score range 8–32), Problem-Solving (4 items, 2 strategies, i.e., active coping and planning, range 4–16), Positive Thinking (6 items, 3 strategies, i.e., positive reframing, acceptance, and humor, range 6–24) and Avoidance (10 items, 5 strategies, i.e., disengagement, self-blame, self-distraction, denial, and substance use, range 10–40) (41). Dimension scores were obtained by summing the respective items and dichotomized into “good” or “bad” coping based on a 62.5% cut-off of the maximum score. Higher scores in Seeking Social Support (≥20), Problem-Solving (≥10), and Positive Thinking (≥15) indicated good coping, while higher Avoidance scores (≥25) reflected maladaptive coping. Coping patterns were further stratified by gender and exposure to workplace harassment (yes/no).

#### 2.2.4 Work ability index (WAI)

The Work Ability Index (WAI) was used to assess capacity to perform work, integrating physical, psychological, and occupational health dimensions. It evaluates seven key aspects: current work ability compared to the individual’s lifetime best, work ability in relation to job demands, the number of diagnosed conditions, extent to which these conditions limit work performance, amount of sick leave taken over the past 12 months, personal prognosis of work ability over the next 2 years, and mental resources, including emotional resilience and stress management (42). The total WAI score is obtained by summing the scores across all seven components, yielding a value between 7 and 49. Based on this score, work ability is classified into four categories: poor (7–27), moderate (28–36), good (37–43), and excellent (44–49) (43). To further examine potential differences in work ability, the data were stratified by gender and by reported exposure to workplace harassment (yes/no).

#### 2.2.5 Statistical analyses

All statistical analyses were performed using GraphPad Prism version 9.0 for Windows (GraphPad Software, La Jolla, CA, USA). Descriptive statistics were reported as means ± standard deviations (SD) for continuous variables, where appropriate. The Shapiro-Wilk test was used to assess the normality of data distribution. Depending on the distribution, comparisons between two groups were conducted using either Student’s t-test for normally distributed data or the Mann-Whitney U test for non-parametric data. For comparisons involving three or more groups, one-way ANOVA or Friedman’s test was applied, as appropriate. Categorical variables were analyzed using Chi-square (χ^2^) tests. To investigate differences in exposure to workplace harassment, analyses were stratified by gender. In addition, work ability, personality traits, and coping strategies were examined by stratifying data according to both gender and reported exposure to workplace harassment (yes/no). Correlations between questionnaire variables were assessed using Spearman’s rank correlation coefficients. Specifically, personality traits (Neuroticism, Extraversion, Openness, Agreeableness, Conscientiousness), coping strategies (Problem-Solving, Seeking Social Support, Positive Thinking, Avoidance), and work ability index dimensions were analyzed. To evaluate the relationships between distance matrices, the Mantel test was further employed. Corresponding correlograms were generated using ChiPlot (44) (last accessed 03 June 2025). Statistical significance was set at *p* < 0.05. Results were further annotated according to conventional thresholds: *p* < 0.05 (*), *p* < 0.01 (**), *p* < 0.001 (***), and *p* < 0.0001 (****).

#### 2.2.6 Ethics statement

The study was conducted in accordance with the Declaration of Helsinki and approved by the Ethics Committee of the “G. Martino” University Hospital in Messina no. 0005156/2024.

## 3 Results

### 3.1 Sociodemographic data, job features and harassment questionnaire results highlight gender differences

The sample comprised 415 workers, including 138 men (33.3%) and 277 women (66.7%), with an effective participation rate of 94.97% among those who accessed the survey. Table 1 details the study population. Men were mainly aged 30–49 (55.8%, *n* = 77), while women predominated in the 19–29 (33.2% vs. 28.3%) and 50–60 + (26.0% vs. 15.9%) groups. Educational level differed by gender (*p* = 0.021), with more men holding postgraduate degrees (34.1% vs. 22.7%). More men reported no children (73.9% vs. 59.2%), and women more often had two children (18.8% vs. 8.0%) (*p* = 0.009). No significant gender differences appeared for psychological disorders (*p* = 0.853) or psychiatric diagnoses (*p* = 0.093).

**Table 1:** Socio-demographic characteristics and perception of violence.

Feature	Total	Males	Females	χ^2^	*p*-value
	*n* (%)	*n* (%)	*n* (%)		
Sample	415 (100)	138 (33.3)	277 (66.7)	nd	nd
Age (years)				9.352	0.009
19 to 29	131 (31.6)	39 (28.3)	92 (33.2)		
30 to 49	190 (45.8)	77 (55.8)	113 (40.8)		
50 to 60+	94 (22.7)	22 (15.9)	72 (26.0)		
Education				9.645	0.021
Middle school	2 (0.5)	1 (0.7)	1 (0.4)		
High School	64 (15.4)	13 (9.4)	51 (18.4)		
Graduate	239 (57.6)	77 (55.8)	162 (58.5)		
Postgraduate	110 (26.5)	47 (34.1)	63 (22.7)		
Marital Status				5.507	0.138
Single	224 (54.0)	82 (59.4)	142 (51.3)		
Partner	169 (40.7)	53 (38.4)	116 (41.9)		
Separated	19 (4.6)	3 (2.2)	16 (5.8)		
Widower	3 (0.7)	0 (0.0)	3 (1.1)		
Children				13.474	0.009
0	266 (64.1)	102 (73.9)	164 (59.2)		
1	56 (13.5)	17 (12.3)	39 (14.1)		
2	63 (15.2)	11 (8.0)	52 (18.8)		
3	20 (4.8)	4 (2.9)	16 (5.8)		
>3	3 (0.7)	2 (1.4)	1 (0.4)		
Pregnant (her/partner)	7 (1.7)	2 (1.4)	5 (1.8)		
Miscarriages				nd	nd
Never	351 (84.6)	n.d.	240 (86.6)		
Spontaneous miscarriage	27 (6.5)	n.d.	27 (9.7)		
Induced miscarriage	7 (1.7)	n.d.	7 (2.5)		
Not declared	30 (7.2)	n.d.	3 (1.1)		
Psychological disorder				0.034	0.853
Yes	59 (14.2)	19 (13.8)	40 (14.4)		
No	356 (85.8)	119 (86.2)	237 (85.6)		
Psychological or psychiatric diagnosis				2.805	0.093
Yes	38 (9.2)	8 (5.8)	30 (10.8)		
No	377 (90.8)	130 (94.2)	247 (89.2)		
Victim of harassment				5.428	0.020
Yes	132 (31.8)	33 (23.9)	99 (35.7)		
No	279 (67.2)	102 (73.9)	177 (63.9)		
Not declared	4 (1.0)	3 (2.2)	1 (0.4)		
Been in a violent relationship				6.886	0.075
Yes	80 (19.3)	18 (13.0)	62 (22.4)		
More than once	2 (0.5)	1 (0.7)	1 (0.4)		
No	317 (76.4)	111 (80.4)	206 (74.4)		
Not declared	16 (3.9)	8 (5.8)	8 (2.9)		
Victim of parental violence				6.827	0.033
Yes	53 (12.8)	13 (9.4)	40 (14.4)		
No	345 (83.1)	115 (83.3)	230 (83.0)		
Not declared	17 (4.1)	10 (7.2)	7 (2.5)		

n.d., not determined.

Notably, 35.7% of women (*n* = 99) reported lifetime experiences of workplace harassment, compared to 23.9% of men (*n* = 33); this difference was statistically significant (*p* = 0.020), indicating a higher prevalence among women. Considering only workers who answered to all below-reported questionnaires (*n* = 125), reports of workplace harassment within the past 12 months mirrored lifetime trends, with a significantly higher prevalence among women (36.2%, *n* = 94) compared to men (23.3%, *n* = 31); *χ*^2^ = 6.69, *p* = 0.010. Although more women reported having been involved in a violent intimate partner relationship compared to men (22.4%, *n* = 62 vs. 13.0%, *n* = 18), this difference was not statistically significant (*χ*^2^ = 6.886, *p* = 0.075). Conversely, a significant gender difference was observed in experiences of parental violence (*χ*^2^ = 6.827, *p* = 0.033), with more women (14.4%, *n* = 40) than men (9.4%, *n* = 13) reporting such experiences.

Table 2 summarizes job-related characteristics from the WHHSQ. Most participants were Italian, with no significant gender difference (men: 93.5%, *n* = 129; women: 94.9%, *n* = 263; *p* = 0.310). Men were mainly physicians (67.4%, *n* = 93), whereas women were more frequently nurses (32.1%, *n* = 89 vs. 18.8%, *n* = 26), and midwives were reported only among women (1.8%, *n* = 5). Men more often held resident roles (55.8%, *n* = 77) or senior manager positions (13.8%, *n* = 19), while women were more commonly hospital staff (26.8%, *n* = 74). Regarding work experience, a higher percentage of men reported 1–5 years of seniority (41.3%, *n* = 57 vs. 28.9%, *n* = 80), while more women had over 20 years of experience (20.9%, *n* = 58 vs. 13.8%, *n* = 19). Additional details on employment status, shift work, and workplace settings are available in Table 2.

**Table 2:** Work experience and job features.

Query and answers	Total		Males		Females	
	*N*	%	*N*	%	*N*	%
Did you move from another country to the place where you are currently working?						
NA	5	1.2	1	0.7	4	1.4
Yes	18	4.3	8	5.8	10	3.6
No	392	94.5	129	93.5	263	94.9
If yes. When did you move?						
NA	399	96.1	131	94.9	268	96.8
11 months ago or less	1	0.2	1	0.7	0	0
1–5 years ago	10	2.4	3	2.2	7	2.5
6 years ago or more	5	1.2	3	2.2	2	0.7
Which category best describes your present professional group?						
NA	7	1.7	1	0.7	6	2.2
physician	224	54	93	67.4	131	47.3
nurse	115	27.7	26	18.8	89	32.1
midwife	5	1.2	0	0	5	1.8
pharmacist	1	0.2	0	0	1	0.4
ambulance	0	0	0	0	0	0
auxiliary. ancillary	7	1.7	3	2.2	4	1.4
administration. clerical	6	1.4	3	2.2	3	1.1
professions allied to medicine (therapists. radiographers. assistants)	31	7.5	9	6.5	22	7.9
technical staff (laboratory. sterilization)	3	0.7	0	0	3	1.1
support staff (kitchen. maintenance. security)	2	0.5	1	0.7	1	0.4
other	14	3.4	2	1.4	12	4.3
Which category best describes your present position?						
NA	12	2.9	2	1.4	10	3.6
senior manager	43	10.4	19	13.8	24	8.7
staff	95	22.9	21	15.2	74	26.8
resident	194	46.9	77	55.8	117	42.4
independent	5	1.2	2	1.4	3	1.1
line manager	3	0.7	0	0	3	1.1
other	62	15	17	12.3	45	16.3
How many years of work experience in the health sector do you presently have?						
NA	15	3.6	1	0.7	14	5.1
under 1 year	74	17.8	26	18.8	48	17.3
1–5	137	33	57	41.3	80	28.9
6–10	52	12.5	18	13	34	12.3
11–15	27	6.5	10	7.2	17	6.1
16–20	33	8	7	5.1	26	9.4
over 20 years	77	18.6	19	13.8	58	20.9
In your main job. do you work:						
NA	13	4.8	3	2.2	17	6.1
full-time	269	84.1	116	84.1	233	84.1
part-time	132	5.8	10	7.2	14	5.1
temporary. casual	1	5.3	9	6.5	13	4.7
Do you work in night shifts?						
NA	13	3.1	1	0.7	12	4.3
Yes	270	65.1	92	66.7	178	64.3
No	132	31.8	45	32.6	87	31.4
Where do you spend most of your time (more than 50%) in your main job?						
NA	20	4.8	1	0.7	19	6.9
ambulatory	79	19	35	25.4	44	15.9
general medicine	41	9.9	22	15.9	19	6.9
general surgery	30	7.2	10	7.2	20	7.2
psychiatric	16	3.9	9	6.5	7	2.5
emergency	14	3.4	6	4.3	8	2.9
operating room	1	0.2	0	0	1	0.4
intensive care	32	7.7	9	6.5	23	8.3
management	35	8.4	9	6.5	26	9.4
specialized unit (es. pediatrics. orthopedics. radiology)	21	5.1	6	4.3	15	5.4
technical services (laboratory. sterilization)	117	28.2	30	21.7	87	31.4
support services (kitchen. maintenance)	9	2.2	1	0.7	8	2.9
The number of staff present in the same work setting with you during most (more than 50%) of your work time is:						
NA	15	3.6	2	1.4	13	4.7
none	12	2.9	6	4.3	6	2.2
1–5	180	43.4	65	47.1	115	41.5
6–10	114	27.5	38	27.5	76	27.4
11–15	52	12.5	14	10.1	38	13.7
over 15	42	10.1	13	9.4	29	10.5

NA, Not answering.

Table 3 presents perceptions and experiences related to workplace harassment. Overall, more than half of employees reported being “not at all worried” about harassment (53.0%, *n* = 220), with 60.9% of men (*n* = 84) and 49.1% of women (*n* = 136) expressing no concern. Women were significantly more concerned about the lack of reporting procedures than men, with fewer women (27.8%, *n* = 77) than men (44.2%, *n* = 61) reporting their presence (*p* = 0.0012). When asked whether they felt encouraged to report harassment, the majority of respondents (63.4%, *n* = 263) answered negatively, with no statistically significant gender difference (*p* = 0.2799).

**Table 3:** Perception of harassment and work policies.

Query and answers	Total		Males		Females		*χ* ^2^	*p*-value
	*N*	%	*N*	%	*N*	%		
How worried are you about harassment in your current workplace?							8.3398	0.7989
NA	17	4.1	4	2.9	13	4.7		
not worried at all	220	53	84	60.9	136	49.1		
mildly worried	70	16.9	20	14.5	50	18.1		
moderately worried	49	11.8	9	6.5	40	14.4		
very worried	28	6.7	9	6.5	19	6.9		
extremely worried	31	7.5	12	8.7	19	6.9		
Are there procedures for the reporting of harassment in your workplace?							10.5343	0.00117
NA	20	4.8	5	3.6	15	5.4		
Yes	138	33.3	61	44.2	77	27.8		
No	257	61.9	72	52.2	185	66.8		
Is there encouragement to report workplace harassment?							1.1678	0.2798
NA	11	2.7	3	2.2	8	2.9		
Yes	141	34	52	37.7	89	32.1		
No	263	63.4	83	60.1	180	65		

NA, Not answering.

Table 4 summarizes experiences and perceptions related to verbal abuse in the workplace. Over the past year, 30.4% of participants (*n* = 126) reported being victims of verbal abuse, with men reporting higher rates than women (35.5%, *n* = 49 vs. 27.8%, *n* = 77). Among those affected, 9.4% (*n* = 39) experienced frequent abuse, while 18.1% (*n* = 75) reported occasional episodes. The majority of respondents (67.7%, *n* = 281) reported no incidents. Supervisors were most frequently identified as perpetrators (12.8%, *n* = 53), followed by patients’ family members (6.7%, *n* = 28), patients themselves (4.6%, *n* = 19), and colleagues (3.4%, *n* = 14). Verbal abuse was primarily reported within the healthcare facility (27.5%, *n* = 114), and only one incident (0.2%) occurred outside the workplace. Regarding perceptions, 27.0% (*n* = 112) considered verbal abuse a common issue in their workplace. Emotional consequences included heightened vigilance (3.6%, *n* = 15), disturbing memories (1.9%, *n* = 8), extreme avoidance (1.2%, *n* = 5), and significant fatigue (4.3%, *n* = 18). A quarter of the participants (25.1%, *n* = 104) believed the incident could have been avoided. Institutional responses were reported as limited: 24.3% (*n* = 101) said no investigation followed the event, only 1% (*n* = 4) were offered the chance to discuss or report it, and just 0.5% (*n* = 2) received any additional support. Overall satisfaction with how incidents were managed was low, with 16.1% (*n* = 67) being “very dissatisfied” and only 1.4% (*n* = 6) “very satisfied.”

**Table 4:** Verbal abuse in the workplace.

Query and answers	Total		Males		Females	
	*N*	%	*N*	%	*N*	%
In the last 12 months, have you been verbally abused in your workplace?						
NA	8	1.9	1	0.7	7	2.5
Yes	126	30.4	49	35.5	77	27.8
No	281	67.7	88	63.8	193	69.7
How often have you been verbally abused in the last 12 months?						
NA	291	70.1	91	65.9	200	72.2
all the time	39	9.4	13	9.4	26	9.4
sometimes	75	18.1	29	21.0	46	16.6
once	10	2.4	5	3.6	5	1.8
The last time, who verbally abused you?						
NA	291	70.1	91	65.9	200	72.2
patient/client	19	4.6	3	2.2	16	5.8
relatives of patient/client	28	6.7	12	8.7	16	5.8
staff member	14	3.4	6	4.3	8	2.9
management/supervisor	53	12.8	19	13.8	34	12.3
external colleague/worker	4	1.0	3	2.2	1	0.4
general public	0	0.0	0	0.0	0	0.0
other	6	1.4	4	2.9	2	0.7
Do you consider this to be a typical incident of verbal abuse in your workplace?						
NA	292	70.4	91	65.9	201	72.6
Yes	112	27.0	42	30.4	70	25.3
No	11	2.7	5	3.6	6	2.2
Where did the verbal abuse take place?						
NA	291	70.1	91	65.9	200	72.2
inside health institution or facility	114	27.5	44	31.9	70	25.3
at patient’s/client’s home	0	0.0	0	0.0	0	0.0
outside (on way to work/health visit/home)	1	0.2	0	0.0	1	0.4
other	9	2.2	3	2.2	6	2.2
Since you were abused, how bothered have you been by: Q1: Repeated, disturbing memories, thoughts, or images of the abuse?						
NA	293	70.6	92	66.7	201	72.6
not at all	32	7.7	14	10.1	18	6.5
a little bit	35	8.4	16	11.6	19	6.9
moderately	23	5.5	7	5.1	16	5.8
quite a bit	24	5.8	7	5.1	17	6.1
extremely	8	1.9	2	1.4	6	2.2
Q2: Avoiding thinking about or talking about the abuse or avoiding having feelings related to it?						
NA	295	71.1	92	66.7	203	73.3
not at all	40	9.6	17	12.3	23	8.3
a little bit	38	9.2	16	11.6	22	7.9
moderately	23	5.5	8	5.8	15	5.4
quite a bit	14	3.4	3	2.2	11	4.0
extremely	5	1.2	2	1.4	3	1.1
Q3: Being super-alert or watchful and on guard?						
NA	294	70.8	92	66.7	202	72.9
not at all	15	3.6	10	7.2	5	1.8
a little bit	26	6.3	15	10.9	11	4.0
moderately	34	8.2	12	8.7	22	7.9
quite a bit	28	6.7	3	2.2	25	9.0
extremely	18	4.3	6	4.3	12	4.3
Q4: Feeling like everything you did was an effort?						
NA	295	71.1	92	66.7	203	73.3
not at all	31	7.5	16	11.6	15	5.4
little bit	21	5.1	7	5.1	14	5.1
moderately	21	5.1	11	8.0	10	3.6
quite a bit	29	7.0	7	5.1	22	7.9
extremely	18	4.3	5	3.6	13	4.7
Do you think the incident could have been prevented?						
NA	293	70.6	92	66.7	201	72.6
Yes	104	25.1	37	26.8	67	24.2
No	18	4.3	9	6.5	9	3.2
Was any action taken to investigate the causes of the verbal abuse?						
NA	293	70.6	91	65.9	202	72.9
Yes	8	1.9	2	1.4	6	2.2
No	101	24.3	40	29.0	61	22.0
Do not know	13	3.1	5	3.6	8	2.9
Did your employer or supervisor offer to provide you with counseling						
NA	378	91.1	124	89.9	254	91.7
Yes	0	0.0	0	0.0	0	0.0
No	37	8.9	14	10.1	23	8.3
Did your employer or supervisor offer to provide you with opportunity to speak about/report it						
NA	377	90.8	124	89.9	253	91.3
Yes	4	1.0	1	0.7	3	1.1
No	34	8.2	13	9.4	21	7.6
Did your employer or supervisor offer to provide you with other support						
NA	378	91.1	124	89.9	254	91.7
Yes	2	0.5	0	0.0	2	0.7
No	35	8.4	14	10.1	21	7.6
How satisfied are you with the manner in which the incident was handled?						
NA	295	71.1	93	67.4	202	72.9
very dissatisfied	67	16.1	25	18.1	42	15.2
dissatisfied	25	6.0	7	5.1	18	6.5
average	16	3.9	10	7.2	6	2.2
satisfied	6	1.4	2	1.4	4	1.4
very satisfied	6	1.4	1	0.7	5	1.8

NA, Not answering.

Table 5 summarizes workplace experiences of bullying and mobbing. In the past year, 17.1% of employees (*n* = 71) reported being victims, including 15.2% of men (*n* = 21) and 18.1% of women (*n* = 50), while 80.7% (*n* = 335) reported no such incidents. Among those affected, 5.3% (*n* = 22) experienced it frequently, 10.4% (*n* = 43) occasionally, and 1.4% (*n* = 6) only once. Supervisors were most commonly identified as perpetrators (9.9%, *n* = 41), followed by colleagues (5.8%, *n* = 24). Isolated cases involved patients or relatives (0.5% each, *n* = 2). Around 15.9% (*n* = 66) considered bullying/mobbing a common issue in their workplace, with little gender variation. Most episodes occurred within the healthcare setting (15.9%, *n* = 66), and only 0.7% (*n* = 3) occurred outside. Emotional impact was present but limited: 3.1% (*n* = 13) reported disturbing memories, 1.9% (*n* = 8) avoidance, 4.6% (*n* = 19) heightened vigilance, and 3.6% (*n* = 15) fatigue with daily tasks. Most (14.9%, *n* = 62) believed the event could have been avoided. Institutional responses were often lacking, with 15.4% (*n* = 64) confirming that no investigation followed, and 4.8% (*n* = 20) reported no psychological support (5.8% women vs. 2.9% men). Only 1.0% (*n* = 4) were “very satisfied” with how the incident was managed, compared to 11.8% (*n* = 49) who were “very dissatisfied.”

**Table 5:** Bullying or mobbing in the workplace.

Query and answers	Total		Males		Females	
	*N*	%	*N*	%	*N*	%
In the last 12 months, have you been bullied/mobbed in your workplace?						
NA	9	2.2	3	2.2	6	2.2
Yes	71	17.1	21	15.2	50	18.1
No	335	80.7	114	82.6	221	79.8
How often have you been bullied/mobbed in the last 12 months?						
NA	344	82.9	117	84.8	227	81.9
all the time	22	5.3	6	4.3	16	5.8
sometimes	43	10.4	13	9.4	30	10.8
once	6	1.4	2	1.4	4	1.4
The last time, who bullied/mobbed you?						
NA	344	82.9	117	84.8	227	81.9
patient/client	2	0.5	1	0.7	1	0.4
relatives of patient/client	2	0.5	0	0.0	2	0.7
staff member	24	5.8	10	7.2	14	5.1
management/supervisor	41	9.9	10	7.2	31	11.2
external colleague/worker	0	0.0	0	0.0	0	0.0
general public	0	0.0	0	0.0	0	0.0
other	2	0.5	0	0.0	2	0.7
Do you consider this to be a typical incident of bullying/mobbing in your workplace?						
NA	345	83.1	118	85.5	227	81.9
Yes	66	15.9	19	13.8	47	17.0
No	4	1.0	1	0.7	3	1.1
Where did the bullying/mobbing take place?						
NA	344	82.9	117	84.8	227	81.9
inside health institution or facility	66	15.9	19	13.8	47	17.0
at patient’s/client’s home	0	0.0	0	0.0	0	0.0
outside (on way to work/health visit/home)	3	0.7	2	1.4	1	0.4
other	2	0.5	0	0.0	2	0.7
Since you were abused, how bothered have you been by: Q1: Repeated, disturbing memories, thoughts, or images of the abuse?						
NA	346	83.4	117	84.8	229	82.7
not at all	8	1.9	2	1.4	6	2.2
a little bit	19	4.6	5	3.6	14	5.1
moderately	17	4.1	7	5.1	10	3.6
quite a bit	12	2.9	3	2.2	9	3.2
extremely	13	3.1	4	2.9	9	3.2
Q2: Avoiding thinking about or talking about the abuse or avoiding having feelings related to it?						
NA	347	83.6	117	84.8	230	83.0
not at all	15	3.6	4	2.9	11	4.0
a little bit	24	5.8	8	5.8	16	5.8
moderately	9	2.2	2	1.4	7	2.5
quite a bit	12	2.9	4	2.9	8	2.9
extremely	8	1.9	3	2.2	5	1.8
Q3: Being super-alert or watchful and on guard?						
NA	347	83.6	118	85.5	229	82.7
not at all	7	1.7	3	2.2	4	1.4
a little bit	12	2.9	4	2.9	8	2.9
moderately	20	4.8	4	2.9	16	5.8
quite a bit	10	2.4	2	1.4	8	2.9
extremely	19	4.6	7	5.1	12	4.3
Q4: Feeling like everything you did was an effort?						
NA	346	83.4	117	84.8	229	82.7
not at all	7	1.7	3	2.2	4	1.4
a little bit	8	1.9	3	2.2	5	1.8
moderately	22	5.3	4	2.9	18	6.5
quite a bit	17	4.1	5	3.6	12	4.3
extremely	15	3.6	6	4.3	9	3.2
Do you think the incident could have been prevented?						
NA	346	83.4	118	85.5	228	82.3
Yes	62	14.9	18	13.0	44	15.9
No	7	1.7	2	1.4	5	1.8
Was any action taken to investigate the causes of the bullying/mobbing?						
NA	345	83.1	117	84.8	228	82.3
Yes	3	0.7	2	1.4	1	0.4
No	64	15.4	19	13.8	45	16.2
Do not know	3	0.7	0	0.0	3	1.1
Did your employer or supervisor offer to provide you with counseling						
NA	395	95.2	134	97.1	261	94.2
Yes	0	0.0	0	0.0	0	0.0
No	20	4.8	4	2.9	16	5.8
Did your employer or supervisor offer to provide you with opportunity to speak about/report it						
NA	394	94.9	134	97.1	260	93.9
Yes	2	0.5	0	0.0	2	0.7
No	19	4.6	4	2.9	15	5.4
Did your employer or supervisor offer to provide you with other support						
NA	395	95.2	134	97.1	261	94.2
Yes	2	0.5	0	0.0	2	0.7
No	18	4.3	4	2.9	14	5.1
How satisfied are you with the manner in which the incident was handled?						
NA	347	83.6	118	85.5	229	82.7
very dissatisfied	49	11.8	14	10.1	35	12.6
dissatisfied	11	2.7	4	2.9	7	2.5
average	4	1.0	1	0.7	3	1.1
satisfied	0	0.0	0	0.0	0	0.0
very satisfied	4	1.0	1	0.7	3	1.1

NA, Not answering.

Supplementary Table S1 summarizes experiences of sexual harassment in the workplace. Over the past 12 months, 2.2% of participants (*n* = 9) reported being victims of sexual harassment. Among men, 3.6% (*n* = 5) reported harassment, compared to 1.4% (*n* = 4) of women. Most respondents who experienced harassment indicated it occurred only once (1.4%, *n* = 6), with very few reporting frequent incidents. Patients and supervisors were the most commonly identified perpetrators (0.7%, *n* = 3 each), followed by staff members (0.5%, *n* = 2). The majority of harassment took place within healthcare institutions (1.7%, *n* = 7). Regarding psychological impact, 0.7% of men (*n* = 1) reported being extremely disturbed by memories of the incident, while no women reported similar distress. Avoidance of thoughts or discussions about the harassment was more frequent among men (1.4%, *n* = 2) than women (0.7%, *n* = 1), as was heightened vigilance (men 1.4%, *n* = 2; women 0.4%, *n* = 1). Most respondents expressed dissatisfaction with how their employer or supervisors managed the incident, including the actions taken afterward, the availability of counseling, opportunities to report or discuss the event, and the support offered. Overall, dissatisfaction with the handling of the incident was notably high.

Racial harassment was rare in the sample, with only one male employee reporting an incident in the past year. This incident occurred frequently, was attributed to a supervisor, and took place within the healthcare facility. The respondent perceived this behavior as a common issue in the workplace. Psychological distress was minimal, though the incident was considered preventable. No investigation was conducted, and satisfaction with how the incident was managed was very low (Supplementary Table S2).

**Figure 1:**
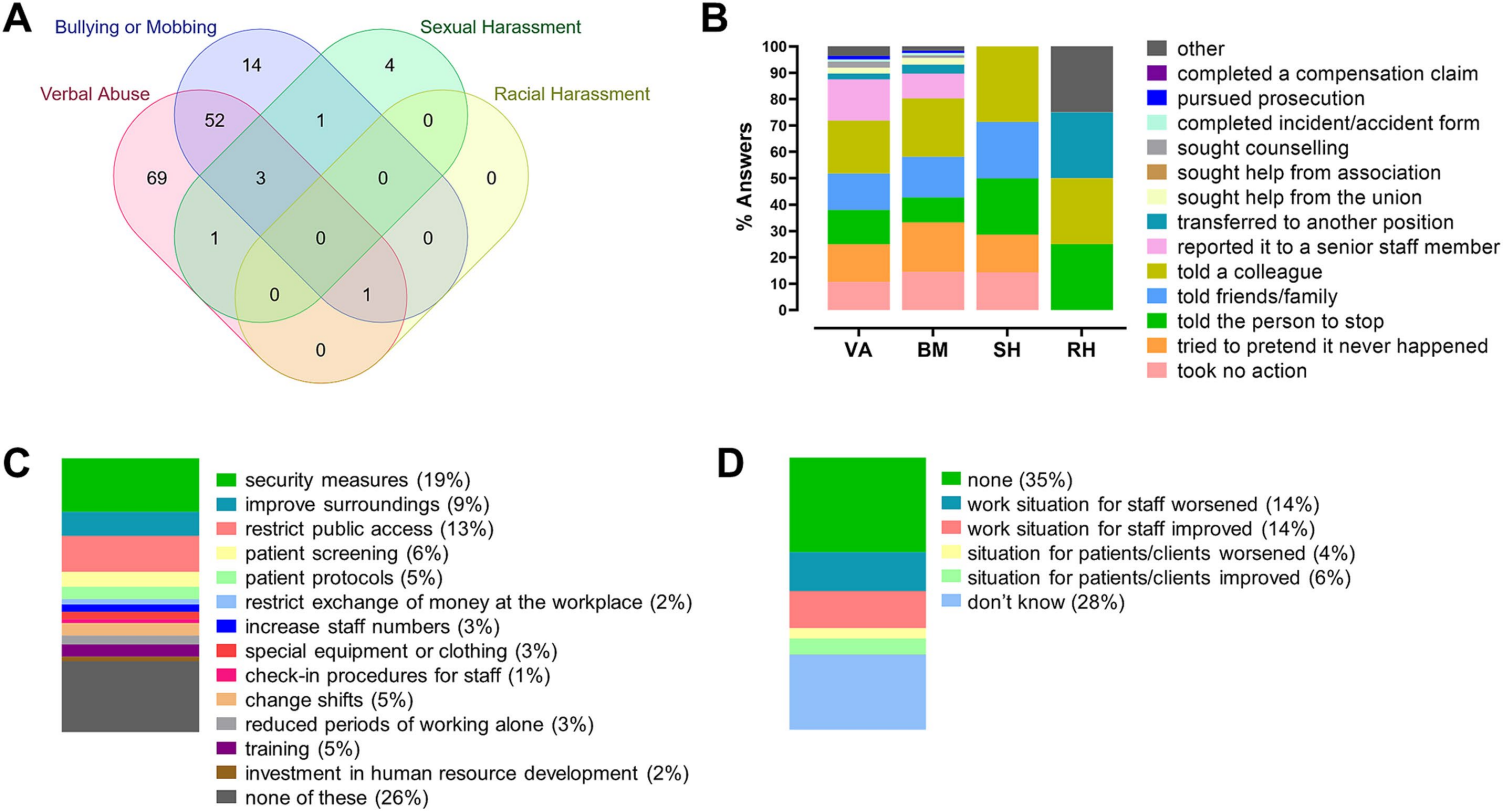
Reactions to workplace harassment. **(A)** Venn diagram illustrating the distribution of worker responses to different types of workplace harassment. **(B)** Bar chart showing the percentage of individuals reporting various reactions to specific forms of harassment: verbal abuse (VA; 124 workers provided 224 responses), bullying or mobbing (BM; 71 workers provided 117 responses), sexual harassment (SH; 9 workers provided 14 responses), and racial harassment (RH; 1 worker provided 4 responses). **(C)** Bar chart displaying the frequency of preventive measures reported by workers (*n* = 617 total responses from 379 participants). **(D)** Bar chart summarizing workers’ perceptions of the impact of these measures on daily work (*n* = 406 responses from 378 participants).

Overall, the WHHSQ outcomes reveal a complex overlap of different forms of WPH. A total of 145 healthcare workers (35%) reported experiencing workplace harassment in the past 12 months, with verbal abuse and bullying/mobbing as the most frequently co-occurring forms (56 responders, 14%, Figure 1A). Responses to harassment varied by type of abuse. Among 124 workers who experienced verbal abuse, 224 responses were recorded. The most common reaction was informing a colleague (20.1%), followed by reporting to a senior colleague (15.6%) and confiding in friends or family (13.8%). A total of 10.7% took no action, while 14.3% ignored the incident. Formal responses were rare: 2.2% sought union support, 0.9% filed a formal complaint, and 1.3% took legal action.

Generally, healthcare workers tended to adopt informal strategies such as peer support or ignoring the incident, rather than formal reporting. In cases of bullying or mobbing, 71 workers provided 117 responses. Informing a colleague was again the most frequent action (22.2%), followed by ignoring the incident (18.8%) and taking no action (14.5%). Reporting to supervisors occurred less often (9.4%), and 2.6% sought union help. Filing reports or legal action were each reported by only 0.9%. Regarding sexual harassment, 9 workers provided 14 responses. The most common reactions were informing a colleague (28.6%) and confiding in friends or family (21.4%). Similarly, 21.4% reported telling the perpetrator to stop, while 14.3% ignored or pretended the incident did not occur. No formal actions (such as reporting to supervisors or legal proceedings) were reported. For racial harassment, one worker provided four responses: confronting the perpetrator (25%), informing a colleague (25%), reassignment to a different role (25%), and selecting “other” (25%). No formal actions were taken (Figure 1B).

Workers also reported on workplace measures to prevent harassment and their perceived impact. Among 379 respondents, a total of 617 measures were identified (Figure 1C). The most frequently mentioned were security measures (e.g., guards, alarms, mobile phones) at 19%, followed by restricted public access (13%) and improvements to the work environment (9%). Other interventions included patient-related measures such as aggression assessment (6%) and preventive protocols (5%). Less common were staff increases (3%), special equipment or protective clothing (3%), staff check-in procedures (1%), and human resources development (2%). Notably, 26% reported no preventive measures in their workplace. When asked about the impact of these measures on daily work, 378 workers provided 406 responses (Figure 1D). Among them, 35% stated no impact, while 28% did not know the effect. A total of 14% perceived worsening conditions for staff, and 14% noted improvements. Impact on patients was less frequently mentioned: 4% reported a negative effect, and 6% a positive one. These findings suggest that although some harassment prevention measures exist, their effectiveness and impact remain unclear to many healthcare workers.

### 3.2 Mini-IPIP results demonstrate gender and workplace harassment-related differences in personality trait profiles

**Figure 2:**
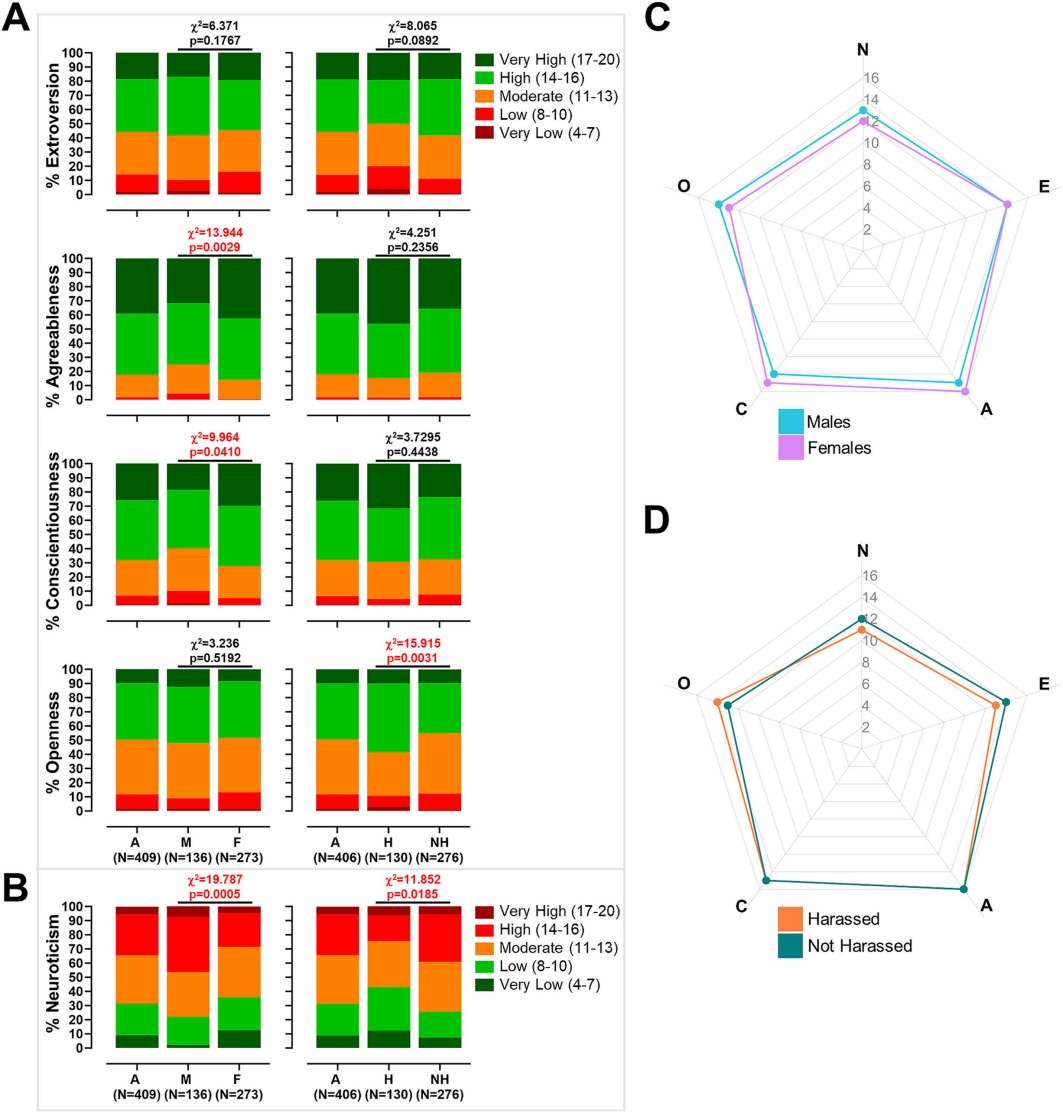
Personality trait distributions and comparisons by gender and workplace harassment. **(A)** Bar plots of extroversion, agreeableness, conscientiousness, and openness scores for the total sample (A), males (M), and females (F) on the left, and for the total sample (A), harassed (H), and not harassed (NH) groups on the right. **(B)** Bar plots of neuroticism scores for the total sample (A), males (M), and females (F) on the left, and for the total sample (A), harassed (H), and not harassed (NH) groups on the right. Chi-square statistics and associated *p*-values are reported on all bar plots to indicate significance of group differences. **(C,D)** Radar plots of the five personality dimensions comparing males versus females **(C)** and harassed versus not harassed individuals **(D)**.

In the total sample, N varied slightly across measures (e.g., *N* = 409 for some personality traits and *N* = 406 for others due to a few missing responses). Mean scores on the five personality dimensions indicated moderately high Extraversion (Mean = 13.9, SD = 3.0), Agreeableness (Mean = 15.9, SD = 2.4), Conscientiousness (Mean = 14.7, SD = 2.8), and Openness to Experience (Mean = 13.4, SD = 2.6), alongside moderate Neuroticism (Mean = 12.0, SD = 3.1). When categorized into five predefined levels (Very Low, Low, Moderate, High, Very High), the majority of participants fell within the Moderate to High ranges across traits. Notably, over 80% of the sample were classified as High or Very High in Agreeableness, and nearly 70% in Conscientiousness. Neuroticism showed the largest variability, with more than 30% of respondents categorized as Low or Very Low, and only 5.6% classified as Very High. These distributions are illustrated in Figure 2 and detailed in Supplementary Tables S3, S4.

Gender-stratified analysis (Males: N = 136; Females: *N* = 273) revealed significant differences in several traits. Females scored significantly higher than males in Agreeableness (Mean = 16.26 vs. 15.29, *p* < 0.001) and Conscientiousness (Mean = 15.03 vs. 14.01, *p* < 0.001), and significantly lower in Neuroticism (Mean = 11.56 vs. 12.93, *p* < 0.001), while no significant differences emerged in Extraversion or Openness. Chi-square analyses of categorical distributions indicated that females were significantly more likely to be categorized as Very High in Agreeableness (42.5% vs. 31.6%, *χ*^2^ = 13.944, *p* = 0.003) and Conscientiousness (29.7% vs. 18.4%, *χ*^2^ = 9.964, *p* = 0.041). In contrast, males were overrepresented in the Moderate and High categories of Neuroticism (70.6% vs. 59.3%, *χ*^2^ = 19.787, *p* < 0.001). No significant gender differences were observed in the categorical distributions of Extraversion or Openness (Figure 2; Supplementary Table S3).

When stratified by exposure to workplace harassment (Harassed: *N* = 130; Not Harassed: *N* = 276), distinct patterns emerged. Participants who reported harassment exhibited significantly lower mean scores in Extraversion (Mean = 13.38 vs. 14.15, *p* = 0.005) and higher scores in Neuroticism (Mean = 12.37 vs. 11.28, *p* < 0.001), with no significant differences in Agreeableness, Conscientiousness, or Openness at the continuous level. Categorical analyses partially corroborated these results, indicating that harassed individuals were more frequently classified as high in Openness (48.5% vs. 35.1%, *χ*^2^ = 15.915, *p* = 0.0031) and as low in Neuroticism (30.8% vs. 18.5%, *χ*^2^ = 11.852, *p* = 0.0185). However, no significant differences were found in the categorical distributions of Extraversion, nor Agreeableness, or Conscientiousness. These results are summarized in Figure 2 and detailed in Supplementary Table S4.

### 3.3 Brief-COPE results demonstrate that coping strategies differ by gender and harassment experience

Analysis of Brief-COPE responses revealed distinct trends in coping strategy use among healthcare workers (*N* = 401). Across the overall sample, coping strategies were employed at moderate levels across all four dimensions, according to established interpretive thresholds. The mean score for Seeking Social Support was 17.20, below the ≥20 threshold for good coping, with moderate use observed across its subcomponents. Problem-Solving strategies were more frequently utilized (*M* = 11.54), exceeding the ≥10 cut-off. Positive Thinking scored slightly below the ≥15 threshold (*M* = 14.11), indicating moderate engagement. Avoidance strategies averaged 18.48, below the ≥25 threshold indicative of maladaptive coping, with higher scores observed in self-blame and self-distraction and lower scores in denial and substance use (see Supplementary Table S5).

As also detailed in Supplementary Table S5, gender-stratified analyses revealed specific patterns in coping strategy use. Females demonstrated significantly greater engagement in Seeking Social Support strategies compared to males. They reported higher levels of emotional support (*M* = 4.45 vs. 4.09, *p* = 0.0086), instrumental support (*M* = 4.72 vs. 4.30, *p* = 0.0017), venting (*M* = 4.54 vs. 4.14, *p* = 0.0043), and religious coping (*M* = 4.09 vs. 3.49, *p* = 0.0014), resulting in a higher overall score in this dimension (*M* = 17.80 vs. 16.02, *p* < 0.00001). However, both groups’ mean scoring remained below the ≥20 threshold indicative of good coping.

**Figure 3:**
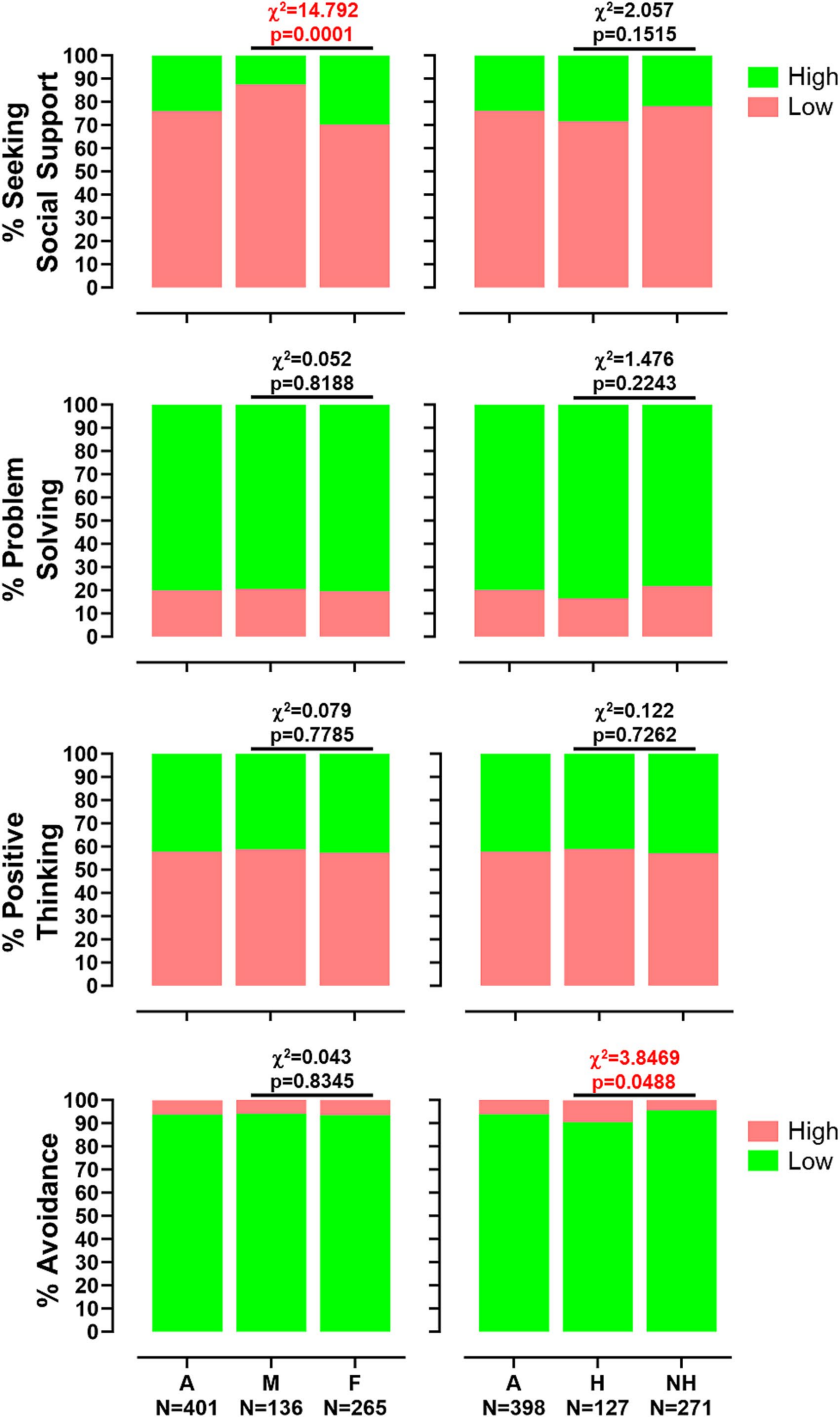
Distribution of coping strategy use from the Brief-COPE questionnaire. Box plots showing the percentage of responders classified as high or low users for the four main coping dimensions: seeking social support, problem-solving, positive thinking, and avoidance. The chart is stratified by gender (men [M] and women [F], left panel) and by experience of workplace harassment (not harassed [NH] and harassed [H], right panel). Chi-squared values and p-values are reported.

No significant gender differences were found for the Problem-Solving dimension (*M* = 11.42 vs. 11.60, *p* = 0.462), with both genders exceeding the ≥10 threshold. Similarly, Positive Thinking mean scores were comparable (*M* = 14.08 vs. 14.13, *p* = 0.880), though still below the ≥15 cut-off. In the Avoidance dimension, no overall gender difference was detected (*M* = 18.16 vs. 18.65, *p* = 0.215). Nevertheless, subscale-level analyses indicated that males reported significantly more substance use (*M* = 2.48 vs. 2.27, *p* = 0.0226), while females reported greater use of self-distraction (*M* = 4.80 vs. 4.45, *p* = 0.0119) and denial (*M* = 3.06 vs. 2.81, *p* = 0.0448). Despite these differences, neither group approached the ≥25 threshold for maladaptive coping. Chi-square analysis confirmed that high use of Seeking Social Support was significantly more frequent among females than males (*χ*^2^ = 14.79, *p* = 0.00012). No significant gender differences were found in the other dimensions (Figure 3).

Coping profiles also differed between participants who reported workplace harassment (*N* = 127) and those who did not (*N* = 271). Both groups demonstrated medium-range scores across most dimensions. No significant differences were observed for Seeking Social Support (*M* = 17.22 vs. 17.18, *p* = 0.924), with similar scores across all subcomponents, including emotional support, instrumental support, venting, and religion. In the Problem-Solving dimension, harassed individuals reported slightly higher scores (*M* = 11.83 vs. 11.41), although this difference was not statistically significant (*p* = 0.0977). Both groups exceeded the ≥10 cut-off for effective coping. Likewise, no significant group differences emerged in Positive Thinking (*M* = 14.16 vs. 14.10, *p* = 0.848), with comparable levels of positive reframing, acceptance, and humor. Both groups remained below the ≥15 threshold.

Notably, Avoidance strategies were significantly more prevalent among participants who reported harassment (*M* = 19.15 vs. 18.16, *p* = 0.0131). Harassed individuals reported higher use of self-distraction (*p* = 0.0166) and substance use (*p* = 0.0417). Although mean scores in both groups remained below the ≥25 threshold for maladaptive coping, the trend suggests increased reliance on avoidant strategies among harassed workers (Supplementary Table S5). This pattern was supported by chi-square analysis, which confirmed a significant difference in Avoidance scores (*χ*^2^ = 3.85, *p* = 0.0498). No significant group differences were observed for Seeking Social Support (*χ*^2^ = 2.06, *p* = 0.151), Problem-Solving (*χ*^2^ = 1.48, *p* = 0.224), or Positive Thinking (*χ*^2^ = 0.12, *p* = 0.726), although harassed participants showed slightly higher mean scores in humor and acceptance (Figure 3).

### 3.4 WAI questionnaire highlights differences based on gender and the harassment experienced

**Figure 4:**
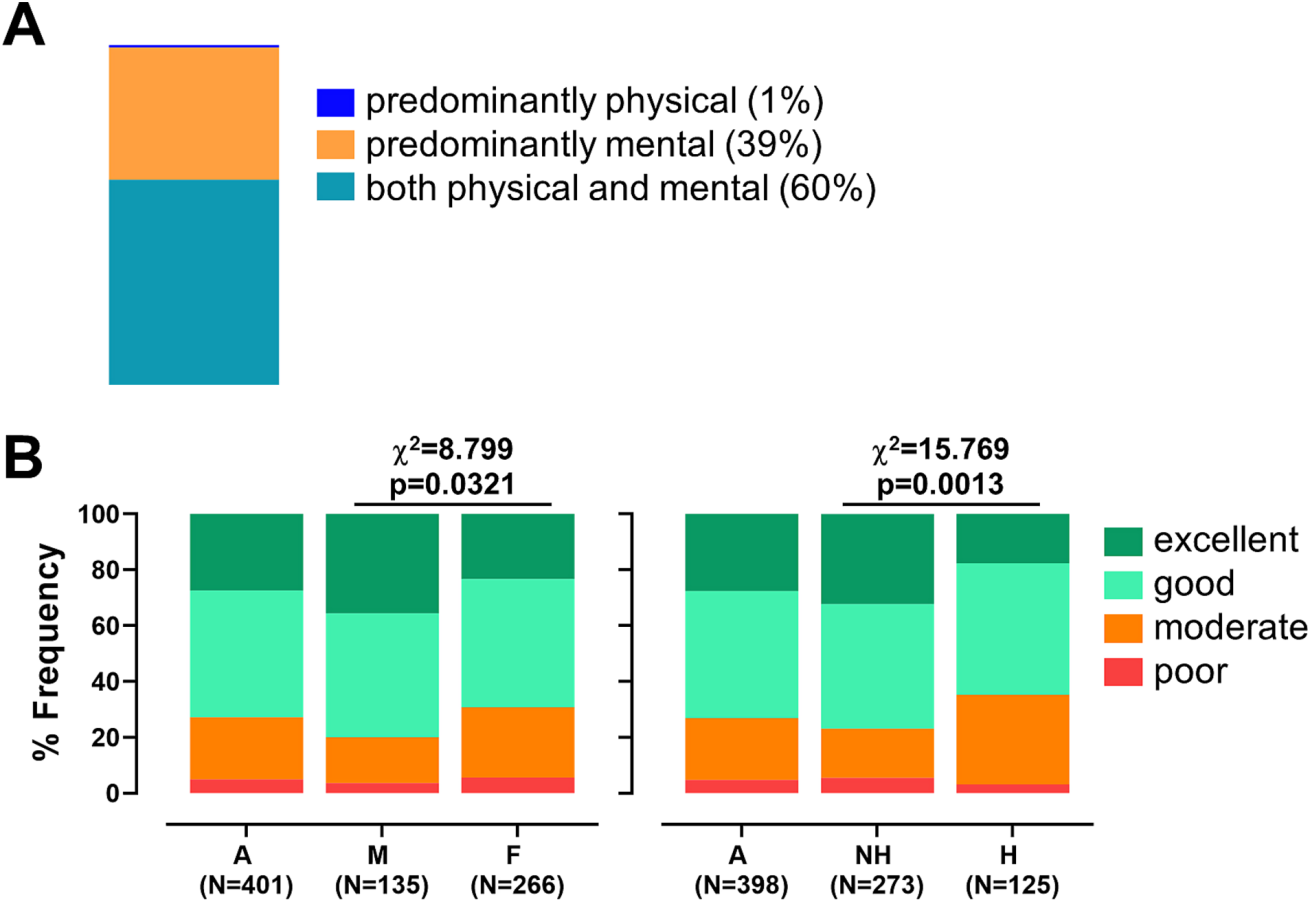
Work characteristics and work ability among healthcare workers. **(A)** Bar chart illustrating the type of work reported by respondents, categorized as predominantly mental, predominantly physical, or a combination of both. **(B)** Bar chart showing the distribution of overall Work Ability Index (WAI) scores, classified as excellent, good, moderate, or poor. The chart is stratified by gender (men [M] and women [F], left panel) and by experience of workplace harassment (not harassed [NH] and harassed [H], right panel). Chi-squared values and p-values are reported.

Out of the 396 responders, 154 (38.9%) reported that their job is predominantly mental, 3 (0.8%) indicated it is mainly physical, while the majority described their work as involving both mental and physical effort (239 workers, 60.3%, Figure 4A).

Work Ability Index (WAI) scores were analyzed by gender (135 men and 266 women, Supplementary Table S6). On average, men reported a significantly higher total WAI score (*M* = 40.21, SD = 5.85) than women (*M* = 38.17, SD = 7.61; *p* = 0.006). As shown in Supplementary Table S6, further analysis of WAI components revealed that men scored significantly better in job demands-related work ability (*p* = 0.038), reported fewer physician-diagnosed illnesses (*p* < 0.001), experienced fewer limitations at work due to illness (*p* = 0.008), and had greater mental resources (*p* = 0.043). When stratifying overall WAI scores, 72.8% of the general sample achieved scores within the “good” or “excellent” range. However, a significantly higher percentage of women fell into the “moderate” or “low” categories compared to men (30.8% vs. 20.0%, *χ*^2^ = 8.799, *p* = 0.032, Figure 4B).

As shown in Supplementary Table S7, WAI scores were also stratified according to experiences of workplace harassment. Workers who reported having experienced harassment had a slightly lower mean WAI score (*M* = 38.10) than those who had not (*M* = 39.34), although this difference did not reach statistical significance (*p* = 0.10). However, harassment was significantly associated with lower scores in the third WAI dimension (physician-diagnosed illnesses) with harassed workers reporting a mean score of 3.93 compared to 4.96 among non-harassed peers (*p* < 0.0001). When stratifying total WAI scores (Figure 4B), the percentage of workers falling into the “moderate” or “low” categories was significantly higher among those who experienced harassment in the workplace (35.2% vs. 23.1%, *χ*^2^ = 15.769, *p* = 0.0013), indicating that exposure to workplace harassment may negatively impact perceived work ability and health status.

### 3.5 Correlation analyses revealed distinct patterns in the associations among work ability, coping strategies, and personality traits in harassed healthcare workers

**Figure 5:**
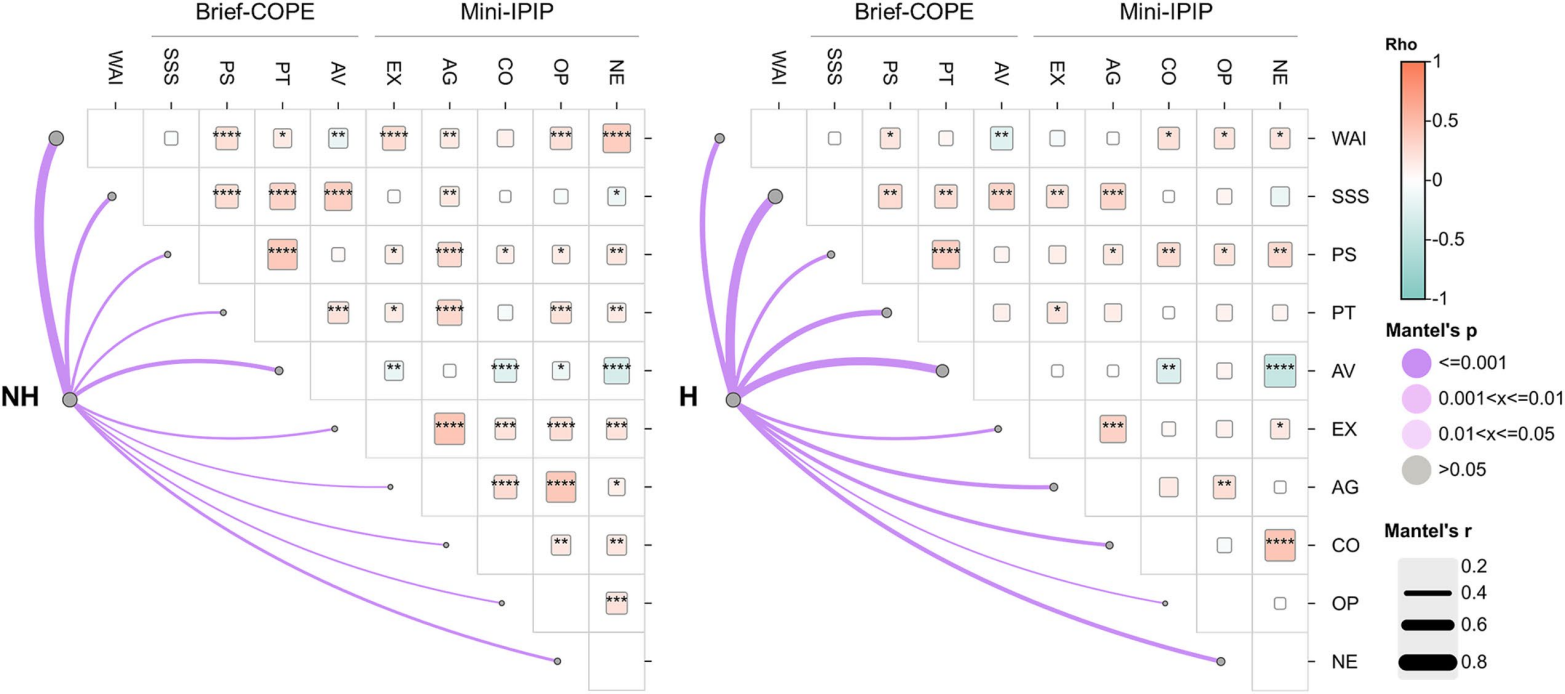
Correlation of work ability, coping strategies, and personality traits in healthcare workers. Correlograms display Spearman correlations and Mantel analysis comparing non-harassed (NH) and harassed (H) healthcare workers. Cell colors represent the direction of correlation: light red for positive (rho between 0 and 1) and light blue for negative (rho between 0 and −1). Square size within each cell is proportional to the magnitude of the rho value. Only statistically significant correlations (*p* < 0.05) are shown. Lines denote Mantel test results, where line width reflects the Mantel r statistic and the intensity of purple shading indicates the significance level (Mantel *p*-value). WAI, Work Ability Index; Brief-COPE: SSS, Seeking Social Support; PS, Problem-Solving; PT, Positive Thinking; AV, Avoidance; Mini-IPIP: EX, Extraversion; AG, Agreeableness; CO, Conscientiousness; OP, Openness; NE, Neuroticism.

Comparative analyses of correlations between key psychological, coping, and work ability measures revealed notable differences between healthcare workers who reported exposure to workplace harassment and those who did not. As shown in Figure 5 and Supplementary Table S8, in the non-harassed group, higher WAI scores were significantly associated with greater levels of Extraversion (*r* = 0.265, *p* < 0.001), Openness (*r* = 0.232, *p* < 0.001), and Agreeableness (*r* = 0.165, *p* = 0.007), and with lower Avoidance coping (*r* = −0.174, *p* = 0.004). Conversely, in the harassed group, WAI was significantly and negatively correlated with Avoidance (*r* = −0.248, *p* = 0.005), but positive associations with personality traits such as Extraversion and Agreeableness were absent. Notably, Conscientiousness correlated positively with WAI only in the harassed group (*r* = 0.226, *p* = 0.011), suggesting a potential protective effect under stress. Social Support Seeking showed moderate-to-strong correlations with Positive Thinking, Problem-Solving and Avoidance in both groups. Additionally, Neuroticism showed a stronger negative correlation with Avoidance in the harassed group (*r* = −0.450, *p* < 0.001) than in the non-harassed group (*r* = −0.315, *p* < 0.001), suggesting a tighter link between emotional instability and maladaptive coping in the presence of workplace harassment.

Mantel tests of matrix correlation further confirmed these patterns. While all Mantel r values were statistically significant (*p* = 0.001), the strength of association between the correlation structures and each target variable varied notably by group. For WAI, the Mantel r was markedly higher in the non-harassed group (*r* = 0.721) than in the harassed group (*r* = 0.401), indicating a more robust and coherent pattern of interrelationships between WAI and the assessed psychological dimensions in the absence of harassment. Conversely, the Mantel r values for coping dimensions, particularly Avoidance (*r* = 0.494), Social Support Seeking (*r* = 0.548), and Positive Thinking (*r* = 0.409), were stronger in the harassed group, suggesting compensatory interdependencies or behavioral adaptations (Figure 5; Supplementary Table S9).

## 4 Discussion

This study comprehensively assessed the prevalence and characteristics of workplace harassment (WPH) among healthcare professionals, focusing on gender differences, personality traits, coping mechanisms, and work ability. The sample consisted of 415 participants, mainly Italians, predominantly female (66%, *n* = 273 vs. male 33%, *n* = 137), with an average age range between 30 and 40 years. Most respondents were physicians (54%), followed by nurses (28%), working full-time in ambulatory settings, with professional seniority between 1 and 5 years. These characteristics reflect a younger, early-career workforce concentrated in clinical frontline roles. Such contexts are known to involve high patient interaction, organizational pressure, and complex team dynamics. These factors are known to potentially heighten vulnerability to interpersonal stressors, including harassment, particularly in hierarchical or poorly managed environments (45).

The findings underscore that WPH is not only prevalent (experienced by 35% of participants in the past year) but also deeply shaped by gendered hierarchies, occupational roles, and institutional inadequacies. Specifically, within the analyzed sample, there was a significantly higher lifetime prevalence of WPH among women (35.7%, *n* = 99) compared to men (23.9%, *n* = 33, *p* = 0.014). Reports of harassment in the past 12 months mirrored these trends, again disproportionately affecting women (36.2% vs. 23.3%, *p* < 0.01).

Remarkably, educational level differed significantly by gender, with a higher percentage of men holding postgraduate degrees (34.1%) compared to women (22.7%, *p* = 0.021). Also, men occupied higher-status roles more frequently, such as physicians (67.4%) and senior managers (13.8%), compared to women (physicians: 47.3%; senior managers: 8.7%), potentially offering greater institutional protection and easier access to compensation mechanisms against WPH, such as legal support, formal grievance procedures, and financial restitution (46). Moreover, women were more often employed as nurses (32.1% vs. 18.8%) or hospital staff (26.8% vs. 15.2%), positions typically involving more direct patient interaction and less structural authority (47). To provide further insight, being underrepresented in leadership might limit women’s institutional power and access to formal support, making it harder to report or address WPH (48). While healthcare male leaders might face stress from managing complex cases, their status often provides them with protection, as WPH against them is less tolerated. Conversely, women in lower-authority roles, despite potentially experiencing less organizational stress, have more direct interpersonal contact (both with patients and colleagues) and less oversight and support, thereby potentially increasing their risk and vulnerability to WPH (49). Job setting may further explain exposure differences: men more often worked in ambulatories (25.4%), where patient interactions are more controlled, while women were more frequently employed in technical services (31.4%), potentially involving less autonomy and greater interpersonal demands, in agreement with what reported by others (50). Altogether, these educational and occupational gender disparities may shape both the risk of exposure to WPH and individuals’ capacity to cope with or report it (51). These results align with broader literature consistently showing higher WPH rates among women in healthcare, likely reflecting persistent gendered hierarchies that position women disproportionately in lower-education, as well as caregiving roles with limited institutional authority (31, 52).

Notably, verbal abuse emerged as the most frequent form of harassment (30.4%), with an unexpected higher prevalence among men (35.5%) than women (27.8%). This inversion compared to prior literature may reflect role-specific dynamics where men, often occupying positions such as residents or frontline physicians, might face high-pressure environments with frequent critical communication that increases the exposure to verbal conflicts (53). Additionally, cultural perceptions that associate “masculinity” with resilience, stoicism, and confrontation may influence what is recognized or reported as verbal aggression, with men potentially overlooking or minimizing the subtle, indirect, or emotionally charged form of abuse, while being more inclined to report overt and confrontational verbal incidents (54).

Supervisors were most frequently identified as perpetrators (12.8%), consistent with previous findings highlighting the critical role of hierarchical power in WPH dynamics, where authority figures such as supervisors or managers may employ their positional power to intimidate, silence, or retaliate against subordinates (55). Such misuse of power underscores the need to more effectively address existing structural imbalances and implement robust accountability mechanisms that go beyond standard reporting procedures (including transparent investigation processes and enforceable sanctions for perpetrators) (56). Indeed, when those in positions of authority misuse their institutional power, it can create environments where WPH is both harder to report and more difficult to remediate, particularly if perpetrators are gatekeepers for career advancement or evaluations. This aligns with previous evidence emphasizing that vertical power imbalances significantly shape WPH risk (55). Also, this finding highlights the need for implementing independent reporting channels and accountability mechanisms to protect vulnerable staff and reduce impunity at higher organizational levels (57). This is particularly important in healthcare settings, where verbal aggression by supervisors is often internalized as a routine aspect of hierarchical control or perceived as a normative response to high-pressure environments, rather than recognized as misconduct (58). Our results also indicated that male respondents reported a higher prevalence of verbal harassment perpetrated by patients’ relatives (8.7% of men vs. 5.8% of women). Although this difference did not reach statistical significance (*χ*^2^ = 6.82, *p* = 0.146), likely due to the limited sample size, the observed trend aligns with prior research highlighting the particular vulnerability of male healthcare workers (especially nurses) to aggression from patients’ families. This increased risk may stem from their frequent involvement in frontline, high-pressure care situations, where they are tasked with enforcing rules, delivering difficult news, or managing emotionally charged interactions (59).

Institutional responses to verbal violence remain markedly insufficient. However, even more alarming is the widespread failure to report incidents. Despite the reported prevalence of WHP, only 0.9% of victims filed formal complaints, and just 1.3% pursued legal action. This extreme underreporting suggests not merely dissatisfaction with how cases are handled but a deeper sense of futility or fear surrounding the reporting process. According to existing data, contributing factors may include lack of trust in institutional procedures, fear of retaliation, concerns over confidentiality, or normalized perceptions of harassment within workplace culture (60). These dynamics might produce a climate of silence where perpetrators (especially those in positions of power) might face limited consequences (61). Satisfaction with employer handling was predictably low: only 1.4% were “very satisfied,” while 16.1% were “very dissatisfied.” This high level of dissatisfaction suggests not just procedural gaps, but a broader failure of institutional accountability. In fact, when responses are perceived as dismissive or ineffective, they erode trust, deter future reporting, and may allow WPH to persist (62). These results align with literature showing widespread mistrust in organizational justice systems and highlight the urgent need for transparent, victim-centered response protocols (63).

Mobbing and bullying were reported by 17.1% of respondents, again largely attributed to supervisors (9.9%). This prevalence underscores a recurring issue of hierarchical abuse, in which power differentials may create environments where inappropriate behaviors go unchecked (64). Although prevalence was similar across genders, emotional impacts were consistent with a pattern of institutional neglect: 15.4% said no investigation followed their complaint, and only 1.0% expressed high satisfaction with the response. These results suggest that beyond direct abuse, prolonged psychological harassment is a common yet unaddressed element of the workplace climate, emphasizing the psychological harm of chronic harassment and the widespread institutional failure to protect workers (65). This is particularly evident in healthcare settings where worker wellbeing is foundational to patients’ service quality (66).

Sexual harassment within the examined sample, though reported at lower rates (2.2%), remains a point of concern. Interestingly, a higher percentage of men (3.6%) than women (1.4%) reported such incidents. The small sample size limits strong conclusions, but this unexpected pattern may reflect underreporting by women due to shame, fear of retaliation, or normalization of inappropriate behaviors (67). Alternatively, male respondents may have interpreted certain encounters as sexually inappropriate in ways that deviate from conventional definitions in the literature (68). Regardless, the complete absence of legal or formal responses by victims underlines not only fear or stigma, but also a profound distrust in official processes, where institutions might be experienced by healthcare workers as inaccessible or even complicit (69).

Racial harassment was reported by only one participant, possibly due to the sample’s demographic homogeneity, as the vast majority (95.5%) were Italian nationals, thereby likely reducing the visibility or incidence of racially motivated mistreatment. However, even in this isolated case, employer response was deemed inadequate, reinforcing concerns about institutional indifference to minority vulnerabilities (70).

Importantly, about 40% of harassed population (*n* = 58) reported experiencing more than one type of harassment. This co-occurrence suggests compounded vulnerability for some workers, where multiple harassment forms intersect, possibly intensifying psychological distress and complicating coping strategies (71). Such intersectionality indicates that WPH cannot be addressed in isolation. Risk analyses must take into account how forms of abuse interact and, hence, create a hostile environment not just episodically, but structurally (72). The tendency for overlapping harassment types may also indicate systemic workplace cultures that tolerate or overlook diverse abusive behaviors, pointing to the necessity for comprehensive, multifaceted interventions rather than isolated policy measures (4).

When asked about perceptions of the workplace’s commitment to preventing and addressing harassment, the majority of respondents expressed skepticism or outright dissatisfaction. About half of the responders reported being “not worried at all” about harassment, suggesting possible underrecognition of risk, thus reflecting denial or a sense of powerlessness rather than genuine safety (36). Notably, only 33.3% acknowledged the existence of formal reporting procedures, with a significant gender difference (44.2% of men versus 27.8% of women, *p* = 0.00117). This suggests not only a communication failure, but potentially a gendered inequity in access to institutional resources. If women are less aware or encouraged to report, then they are doubly disadvantaged: both more likely to be targeted and less likely to be supported (73).

Furthermore, only 34% of harassed sample felt encouraged to report harassment. This indicates systemic gaps in awareness and support, particularly disadvantaging women, who are more frequently targeted by verbal harassment (74). The lack of clear, encouraged reporting mechanisms likely contributes to the extremely low rates of formal complaints observed, perpetuating a culture where harassment remains underreported and insufficiently addressed (75). Despite the high frequency of WPH, preventive strategies appear largely symbolic. Among the 617 reported interventions, the most common were security-related (19%) and restricted access (13%). This disjunction between policy presence and perceived efficacy suggests a failure in either implementation or communication, reflecting what has often been described as the “illusion of protection” offered by weak or ineffective anti-harassment policies (76).

The inclusion of the Mini-IPIP scale offered insights into how personality may shape or mediate harassment experiences. Women scored significantly higher on Agreeableness (*M* = 16.26 vs. 15.29, *p* < 0.001) and Conscientiousness (*M* = 15.03 vs. 14.01, *p* < 0.001), and lower on Neuroticism (*M* = 12.93 vs. 11.56, *p* < 0.0001). In line with WPH questionnaire outcomes, these personality profiles may predispose women to roles with high relational demands, potentially exposing them to more subtle forms of exploitation or expectation for emotional resilience (77). In particular, these differences are consistent with prior research on gendered personality profiles and may partly explain women’s preference for emotionally laborious roles in healthcare, suggesting these traits align with traditionally “feminized” roles that demand empathy, diligence, and emotional labor (78, 79).

Participants who reported WPH had significantly lower Neuroticism (*M* = 11.28 vs. 12.37, *p* < 0.0001) and lower Extraversion (*M* = 13.38 vs. 14.15, *p* < 0.01), suggesting that individuals who are less emotionally reactive and more socially withdrawn may be more vulnerable to harassment or less equipped to respond assertively and, hence, to report the mistreatment (80). Conversely, lower Extraversion may reflect a reduced tendency to assert boundaries or seek interpersonal support, potentially increasing vulnerability to targeting or reducing the likelihood of resistance (81). Interestingly, the association between lower Neuroticism and increased WPH exposure contrasts with recent meta-analyses highlighting higher Neuroticism levels among harassed workers (19). This apparent contradiction may point to different underlying mechanisms, such as the possibility that less neurotic individuals underreport emotional distress or, alternatively, they are perceived as easier targets due to lower emotional expressiveness (22). Notably, our findings align with earlier reports suggesting that workers with neurotic tendencies may be more likely to avoid, rather than confront, hostile environments, making them less visible within the harassment statistics (82). One of the novel contributions of this study, therefore, lies in challenging the prevailing assumption that higher Neuroticism predisposes individuals to victimization (83). Intriguingly, Neuroticism may operate both as antecedents and consequences of WPH, a dynamic that deserves to be deeply explored through future longitudinal designs, to further untangle both causality and adaptive responses over time (23).

Moreover, in non-harassed healthcare workers, Openness to Experience demonstrated a significant positive correlation with work ability (*r* = 0.232, *p* < 0.05), suggesting that individuals who are more open to new ideas and experiences may better adapt to job demands and sustain higher work functioning. This adaptability likely stems from their greater cognitive flexibility and willingness to engage with challenges, qualities that are crucial in dynamic healthcare environments (84). Although this positive association remained statistically significant among workers exposed to harassment (*r* = 0.201, *p* < 0.05), it was notably attenuated. Correspondingly, the Mantel test revealed a strong overall correlation for Openness in both groups (*r* = 0.278 vs. 0.268, *p* = 0.001), yet the slight reduction in the harassed group implies that the psychological stress and strain associated with WPH may partially undermine the protective benefits typically provided by this trait. This erosion may reflect how sustained exposure to hostility and stress is able to diminish even typically resilient traits, highlighting that personality strengths alone are insufficient buffers when the organizational support is lacking (85). Overall, these findings suggest that WPH not only directly compromises wellbeing but also diminishes the resilience and adaptive flexibility that Openness ordinarily supports in challenging work environments (86).

Furthermore, analysis of the Brief-COPE scale revealed distinct gendered and harassment-related coping profiles. Women demonstrated significantly higher scores in Seeking Social Support (*M* = 17.80 vs. 16.02, *p* < 0.00001) across all four subcomponents (venting, emotional, religious, and instrumental). This aligns with broader research suggesting that women are more likely to seek collective coping mechanisms in the face of stress (87). However, both men and women failed to reach the optimal threshold (≥20) for effective use of social support, indicating a need for interventions aimed at boosting help-seeking behaviors (51). This data suggests that structural or cultural barriers (such as stigma, lack of trust, or poor institutional responsiveness) may inhibit help-seeking even when workers are inclined to do so (88). Problem-solving strategies were moderately used across the sample (mean around 11.5) but fell short of optimal levels. Positive thinking strategies also remained underutilized. In line with existing data, this indicates a broader pattern of coping insufficiency, likely stemming from environments where support is limited and stressors are chronic, leaving workers without the psychological or practical means to engage in more constructive coping (34).

Of concern, Avoidance strategies were moderately employed, with notable gender differences: men reported higher substance use (*p* = 0.0226), while women more frequently used denial (*p* = 0.0005) and self-distraction (*p* = 0.0048). Avoidance was also significantly higher among workers who experienced harassment (*M* = 19.15 vs. 18.16, *p* = 0.0131), regardless of gender. Despite its well-documented link to poorer outcomes, the observed preference for avoidant coping suggests a sense of powerlessness among harassed individuals, where Avoidance becomes a default strategy when other avenues (e.g., institutional support, reporting mechanisms) appear ineffective or even risky (89). Also, this trend indicates a reliance on passive or maladaptive coping strategies in response to WPH, which may deepen psychological distress and increase vulnerability to continued mistreatment among both male and female workers (90). These results align with prior research linking avoidant coping with poorer mental health outcomes and prolonged exposure to stressful environments (91).

The WAI assessment reveals significant gender differences: men reported a higher work ability (*M* = 40.21 vs. 38.17; *p* = 0.006). Men also scored significantly better in job demands-related ability (*p* = 0.038), had fewer physician-diagnosed illnesses (*p* < 0.001), experienced fewer work limitations due to illness (*p* = 0.008), and reported greater mental resources (*p* = 0.043). These gendered discrepancies may be rooted in the interplay of multiple factors that cumulatively disadvantage women. These factors include: differential exposure to verbal abuse, societal expectations around emotional labor, and unequal access to formal support (92). Regarding WPH, 35.2% of harassed workers fell into moderate or low work ability categories, significantly higher than the 23.1% among non-harassed workers (*p* = 0.0013). These data, in line with others, suggest that WPH might be associated with poorer health outcomes and reduced work capacity, with women disproportionately affected (93). The broader implication is that WPH is not merely an individual health issue but a structural factor undermining workplace equity and resilience (94).

Overall, experiencing WPH appears to exacerbate physical and mental health challenges, leading to more physician-diagnosed illnesses and greater work limitations (mean score 3.93 vs. 4.96, *p* < 0.0001). This disparity likely reflects the cumulative effects of gendered workplace dynamics, including higher exposure to harassment and potentially fewer resources or support systems to mitigate its consequences (95). Hence, these findings might suggest that WPH not only undermines individual wellbeing, but also threatens workforce productivity and sustainability, particularly for women in healthcare roles (32). This highlights the urgent need for interventions that go beyond reactive measures and aim to shift workplace cultures, making them more inclusive, transparent, and responsive to early signs of psychosocial risk (96).

Correlation analyses further confirmed clear differences in how both personality traits and coping mechanisms may relate to work ability between healthcare workers who experienced harassment and those who did not. In non-harassed workers, work ability was positively linked to Positive coping (*r* = 0.156), while it was negatively linked to Avoidance coping (*r* = −0.174). However, in harassed workers, these positive links disappeared. Avoidance coping had a stronger negative impact on work ability (*r* = −0.248), and Neuroticism was more strongly connected to Avoidance maladaptive coping (*r* = −0.450). This shift implies that under the strain of WPH, the normally protective effect of positive coping becomes insufficient, while maladaptive patterns like Avoidance become more detrimental. It reinforces the concept of an existing “coping threshold” beyond which workers’ psychological resources may fail, necessitating external support to prevent collapse, both in their wellbeing and productivity (97). Also, Mantel test results show key differences between harassed and non-harassed healthcare workers in the relationships among work ability, coping, and personality traits. WAI correlated strongly in the non-harassed group (*r* = 0.721, *p* = 0.001) but much less so in the harassed group (*r* = 0.401, *p* = 0.001), indicating harassment weakens the link between work ability and psychological factors. In agreement with previous reports, this breakdown of internal coherence among psychological traits under pressure suggests that chronic WPH not only affects performance but also fundamentally alters how individuals might regulate their psychological responses to stress at work (32).

Coping strategies like Seeking Social Support (0.548 vs. 0.428) and Avoidance (0.494 vs. 0.418) had stronger correlations in the harassed group, suggesting coping becomes more central under harassment-related stress (98). Personality traits such as Agreeableness (0.355 vs. 0.271) and Conscientiousness (0.337 vs. 0.280) also showed higher correlations in harassed workers than in not harassed ones, possibly reflecting greater reliance on these traits to manage adversity (19). This suggests that under conditions of psychosocial strain, healthcare workers increasingly draw on dispositional traits as compensatory strategies. While potentially adaptive in the short term, over-reliance on these traits without institutional support may eventually lead to either burnout or disengagement (99). In summary, WPH appears to disrupt the usual positive link between work ability and psychological health, while increasing the interdependence of coping and personality factors as workers try to adapt, in line with existing literature. This suggests that WPH not only impairs wellbeing but also reshapes the psychological resources on which workers rely to maintain their function and resilience in challenging environments (100).

This study is not without limitations. First, its cross-sectional design precludes making any causal inferences about the relationships between harassment, personality, and coping. Second, reliance on self-reported data may introduce biases, including recall bias, social desirability, and common method variance (101). Third, while the sample was diverse in profession, it was geographically confined to Italy, which may limit generalizability to other sociocultural contexts. Hence, the low reporting of sexual and racial harassment likely reflects underreporting rather than absence.

In conclusion, this study highlights the pervasive and multifaceted impact of WPH on healthcare professionals, revealing significant gender disparities, compounded psychological stress, and weakened work ability. Women are disproportionately affected, facing higher WPH rates, greater health consequences, and lower work capacity. The findings also show that WPH disrupts the normal positive relationships between personality, coping strategies, and work ability, fostering maladaptive coping and increasing reliance on certain personality traits to manage ongoing stress (102). Institutional shortcomings in prevention, reporting, and response further exacerbate these effects, underscoring the urgent need for comprehensive, gender-sensitive policies and support systems to protect vulnerable healthcare workers and promote a healthier, more equitable work environment (103). Notably, Italy has taken a leading role in workplace harassment protection by ratifying ILO Convention No. 190 in 2021 and updating national laws (Legislative Decree 81/2008 and Law No. 4/2021). However, this study highlights ongoing challenges in effectively implementing these legal frameworks within healthcare settings (3).

Taken together, our findings highlight a critical misalignment between the high prevalence of WPH, the psychological toll it imposes, and the inadequate institutional mechanisms in place to address it. Despite growing awareness and policies, healthcare workplaces remain high-risk environments for harassment, particularly verbal abuse and bullying. Future interventions must go beyond reactive measures to foster proactive, trauma-informed systems that are visible, trustworthy, and culturally responsive. In fact, effective prevention requires both individual resilience and systemic change: beyond training and policies, organizations need comprehensive programs with role-specific harassment education, confidential reporting managed by impartial staff, and psychological support for victims. Moreover, the development of effective gender-sensitive and role-specific training, coupled with leadership accountability, will be of pivotal importance. To propose focus groups to sensitive workers is also a gaining strategy to avoid their employment within more at-risk hospital compartments. Finally, future longitudinal researches should investigate how WPH shapes career trajectories and psychological wellbeing over time, and test interventions aimed at improving coping, resilience, and institutional responsiveness. Integrating these efforts into broader occupational health frameworks can drive lasting cultural change, especially in healthcare.

## Data Availability

The raw data supporting the conclusions of this article will be made available by the authors, without undue reservation.
