# Supplementary material for: Workplace harassment is associated with differences in personality traits, coping strategies and work ability: cross sectional study among healthcare professionals
Source: Front Public Health. 2025 Sep 3;13:1641654. doi: 10.3389/fpubh.2025.1641654 (PMC12441026; doi:10.3389/fpubh.2025.1641654)
Supplement: Supplementary file 1 [file Data_Sheet_1.PDF]

Supplementary Material

Supplementary Table S1. Sexual harassment in the workplace.

	Total		Males		Females	
	N	%	*N*	%	*N*	%
In the last 12 months, have you been sexually harassed in your workplace?						
NA	11	2.7	3	2.2	8	2.9
Yes	9	2.2	5	3.6	4	1.4
No	395	95.2	130	94.2	265	95.7
How often have you been sexually harassed in the last 12 months?						
NA	406	97.8	133	96.4	273	98.6
all the time	1	0.2	1	0.7	0	0.0
sometimes	2	0.5	2	1.4	0	0.0
once	6	1.4	2	1.4	4	1.4
The last time, who sexually harassed you?						
NA	406	97.8	133	96.4	273	98.6
patient/client	3	0.7	1	0.7	2	0.7
relatives of patient/client	0	0.0	0	0.0	0	0.0
staff member	2	0.5	1	0.7	1	0.4
management/supervisor	3	0.7	2	1.4	1	0.4
external colleague/worker	1	0.2	1	0.7	0	0.0
general public	0	0.0	0	0.0	0	0.0
other	0	0.0	0	0.0	0	0.0
Do you consider this to be a typical incident of sexual harassment in your workplace?						
NA	406	97.8	133	96.4	273	98.6
Yes	5	1.2	2	1.4	3	1.1
No	4	1.0	3	2.2	1	0.4
Where did the sexual harassment take place?						
NA	406	97.8	133	96.4	273	98.6
inside health institution or facility	7	1.7	4	2.9	3	1.1
at patient's/client's home	0	0.0	0	0.0	0	0.0
outside (on way to work/health visit/home)	1	0.2	1	0.7	0	0.0
other	1	0.2	0	0.0	1	0.4
Since you were abused, how bothered have you been by:						
Q1: ***Repeated, disturbing memories, thoughts, or images of the abuse?***						
NA	406	97.8	133	96.4	273	98.6
not at all	3	0.7	2	1.4	1	0.4
a little bit	1	0.2	0	0.0	1	0.4
moderately	2	0.5	1	0.7	1	0.4
quite a bit	2	0.5	1	0.7	1	0.4
extremely	1	0.2	1	0.7	0	0.0
Q2: ***Avoiding thinking about or talking about the abuse or avoiding having feelings related to it?***						
NA	406	97.8	133	96.4	273	98.6
not at all	3	0.7	2	1.4	1	0.4
a little bit	1	0.2	1	0.7	0	0.0
moderately	3	0.7	0	0.0	3	1.1

quite a bit	1	0.2	1	0.7	0	0.0
extremely	1	0.2	1	0.7	0	0.0
***Q3:* Being super-alert or watchful and on guard?**						
NA	406	97.8	133	96.4	273	98.6
not at all	2	0.5	2	1.4	0	0.0
a little bit	2	0.5	1	0.7	1	0.4
moderately	1	0.2	0	0.0	1	0.4
quite a bit	1	0.2	0	0.0	1	0.4
extremely	3	0.7	2	1.4	1	0.4
***Q4:* Feeling like everything you did was an effort?**						
NA	406	97.8	133	96.4	273	98.6
not at all	3	0.7	2	1.4	1	0.4
a little bit	2	0.5	1	0.7	1	0.4
moderately	1	0.2	0	0.0	1	0.4
quite a bit	1	0.2	1	0.7	0	0.0
extremely	2	0.5	1	0.7	1	0.4
Do you think the incident could have been prevented?						
NA	406	97.8	133	96.4	273	98.6
Yes	7	1.7	3	2.2	4	1.4
No	2	0.5	2	1.4	0	0.0
Was any action taken to investigate the causes of the sexual harassment?						
NA	406	97.8	133	96.4	273	98.6
Yes	0	0.0	0	0.0	0	0.0
No	9	2.2	5	3.6	4	1.4
Don't know	0	0.0	0	0.0	0	0.0
Did your employer or supervisor offer to provide you with counselling						
NA	411	99.0	135	97.8	276	99.6
Yes	0	0.0	0	0.0	0	0.0
No	4	1.0	3	2.2	1	0.4
Did your employer or supervisor offer to provide you with opportunity to speak about/report it						
NA	411	99.0	135	97.8	276	99.6
Yes	2	0.5	2	1.4	0	0.0
No	2	0.5	1	0.7	1	0.4
Did your employer or supervisor offer to provide you with other support						
NA	411	99.0	135	97.8	276	99.6
Yes	0	0.0	0	0.0	0	0.0
No	4	1.0	3	2.2	1	0.4
How satisfied are you with the manner in which the incident was handled?						
NA	406	97.8	133	96.4	273	98.6
very dissatisfied	5	1.2	2	1.4	3	1.1
dissatisfied	0	0.0	0	0.0	0	0.0
average	1	0.2	1	0.7	0	0.0
satisfied	0	0.0	0	0.0	0	0.0
very satisfied	3	0.7	2	1.4	1	0.4

NA, Not answering.

Supplementary Table S2. Racial harassment in the workplace.

	Total		Males	
	N	%	N	%
In the last 12 months, have you been racially harassed in your workplace?				
NA	9	2.2	1	0.7
Yes	1	0.2	1	0.7
No	405	97.6	136	98.6
How often have you been racially harassed in the last 12 months?				
NA	414	99.8	137	99.3
all the time	1	0.2	1	0.7
sometimes	0	0.0	0	0.0
once	0	0.0	0	0.0
The last time, who racially harassed you?				
NA	414	99.8	137	99.3
patient/client	0	0.0	0	0.0
relatives of patient/client	0	0.0	0	0.0
staff member	0	0.0	0	0.0
management/supervisor	1	0.2	1	0.7
external colleague/worker	0	0.0	0	0.0
general public	0	0.0	0	0.0
other	0	0.0	0	0.0
Do you consider this to be a typical incident of racial harassment in your workplace?				
NA	414	99.8	137	99.3
Yes	1	0.2	1	0.7
No	0	0.0	0	0.0
Where did the racial harassment take place?				
NA	414	99.8	137	99.3
inside health institution or facility	1	0.2	1	0.7
at patient's/client's home	0	0.0	0	0.0
outside (on way to work/health visit/home)	0	0.0	0	0.0
other	0	0.0	0	0.0
Since you were abused, how bothered have you been by:				
***Q1:* Repeated, disturbing memories, thoughts, or images of the abuse?**				
NA	414	99.8	137	99.3
not at all	0	0.0	0	0.0
a little bit	1	0.2	1	0.7
moderately	0	0.0	0	0.0
quite a bit	0	0.0	0	0.0
extremely	0	0.0	0	0.0
Q2:* *Avoiding thinking about or talking about the abuse or avoiding having feelings related to it?				
NA	414	99.8	137	99.3
not at all	1	0.2	1	0.7
a little bit	0	0.0	0	0.0
moderately	0	0.0	0	0.0
quite a bit	0	0.0	0	0.0

extremely	0	0.0	0	0.0
***Q3:* Being super-alert or watchful and on guard?**				
NA	414	99.8	137	99.3
not at all	0	0.0	0	0.0
a little bit	0	0.0	0	0.0
moderately	1	0.2	1	0.7
quite a bit	0	0.0	0	0.0
extremely	0	0.0	0	0.0
***Q4:* Feeling like everything you did was an effort?**				
NA	414	99.8	137	99.3
not at all	1	0.2	1	0.7
a little bit	0	0.0	0	0.0
moderately	0	0.0	0	0.0
quite a bit	0	0.0	0	0.0
extremely	0	0.0	0	0.0
Do you think the incident could have been prevented?				
NA	414	99.8	137	99.3
Yes	1	0.2	1	0.7
No	0	0.0	0	0.0
Was any action taken to investigate the causes of the racial harassment?				
NA	414	99.8	137	99.3
Yes	0	0.0	0	0.0
No	1	0.2	1	0.7
Don't know				
Did your employer or supervisor offer to provide you with counselling				
NA	415	100.0	138	100.0
Yes	0	0.0	0	0.0
No	0	0.0	0	0.0
Did your employer or supervisor offer to provide you with opportunity to speak about/report it				
NA	415	100.0	138	100.0
Yes	0	0.0	0	0.0
No	0	0.0	0	0.0
Did your employer or supervisor offer to provide you with other support				
NA	415	100.0	138	100.0
Yes	0	0.0	0	0.0
No	0	0.0	0	0.0
How satisfied are you with the manner in which the incident was handled?				
NA	414	99.8	137	99.3
very dissatisfied	1	0.2	1	0.7
dissatisfied	0	0.0	0	0.0
average	0	0.0	0	0.0
satisfied	0	0.0	0	0.0
very satisfied	0	0.0	0	0.0

NA, Not answering.

Supplementary Table S3. IPIP Questionnaire with gender stratification.

	E	A	C	O	N	
**A=409**	13,90	15,94	14,69	13,41	12,01	**Mean**
	3,00	2,47	2,76	2,55	3,13	SD
	0,29	0,24	0,27	0,25	0,30	C
**M=136**	**14,04**	**15,29**	**14,01**	**13,63**	**12,93**	**Mean**
	2,86	2,58	2,81	2,50	2,80	SD
	0,48	0,43	0,47	0,42	0,47	C
**F=273**	**13,82**	**16,26**	**15,03**	**13,30**	**11,56**	**Mean**
	3,07	2,35	2,67	2,57	3,20	SD
	0,36	0,28	0,32	0,31	0,38	C
	4,85E-01	***1,73E-04***	***3,60E-04***	*2,15E-01*	***2,62E-05***	*P-value*

A, all; M, Men; F, Females; E, Extroversion; A, Agreeableness; C, Conscientiousness; O, Openness; N, Neuroticism.

Supplementary Table S4. IPIP Questionnaire with experienced harassment stratification.

	E	A	C	O	N	
**A=406**	13,90	15,94	14,70	13,39	12,02	**Mean**
	3,00	2,48	2,75	2,55	3,13	SD
	0,29	0,24	0,27	0,25	0,30	C
**H=130**	**13,38**	**16,30**	**14,97**	**13,72**	**11,28**	**Mean**
	3,25	2,52	2,78	2,59	3,34	SD
	0,55	0,42	0,47	0,43	0,56	C
**NH=276**	**14,15**	**15,76**	**14,57**	**13,24**	**12,37**	**Mean**
	2,85	2,44	2,74	2,52	2,97	SD
	0,34	0,29	0,32	0,30	0,35	C
	4,64E-03	*9,17E-02*	*4,96E-01*	*7,92E-01*	***4,40E-05***	*P-value*

A, all; H, Harassed; NH, Not Harassed; E, Extroversion; A, Agreeableness; C, Conscientiousness; O, Openness; N, Neuroticism.

Supplementary Table S5. Brief-COPE questionnaire results.

		Emotional Support	Instrumental Support	Venting	Religion	Seeking Social Support	Active Coping	Planning	Problem Solving	Positive Reframing	Acceptance	Humor	Positive Thinking	Disengagement	Self-Blame	Self-Distraction	Denial	Substance Use	Avoidance
All (N=401)	Mean	4,33	4,58	4,40	3,89	17,20	5,75	5,80	11,54	4,84	5,32	3,95	14,11	3,14	5,34	4,68	2,98	2,34	18,48
	SD	1,32	1,28	1,32	1,77	3,84	1,35	1,40	2,38	1,42	1,37	1,29	2,96	1,09	1,35	1,33	1,21	0,87	3,72
Male (N=136)	Mean	4,09	4,30	4,14	3,49	16,02	5,55	5,87	11,42	4,68	5,32	4,08	14,08	3,20	5,23	4,45	2,81	2,48	18,16
	SD	1,27	1,12	1,21	1,54	3,65	1,38	1,42	2,31	1,44	1,42	1,38	3,14	1,20	1,31	1,34	1,29	1,11	4,22
Female (N=265)	Mean	4,45	4,72	4,54	4,09	17,80	5,85	5,76	11,60	4,92	5,32	3,88	14,13	3,11	5,40	4,80	3,06	2,27	18,65
	SD	1,33	1,34	1,36	1,84	3,80	1,32	1,39	2,41	1,40	1,35	1,24	2,87	1,03	1,37	1,31	1,15	0,72	3,44
	P-value	*8,55E-03*	*1,65E-03*	*4,30E-03*	*1,37E-03*	*9,05E-06*	*3,88E-02*	*4,61E-01*	*4,62E-01*	*1,08E-01*	*9,54E-01*	*1,39E-01*	*8,80E-01*	*4,59E-01*	*2,18E-01*	*1,19E-02*	*4,48E-02*	*2,26E-02*	*2,15E-01*

		Emotional Support	Instrumental Support	Venting	Religion	Seeking Social Support	Active Coping	Planning	Problem Solving	Positive Reframing	Acceptance	Humor	Positive Thinking	Disengagement	Self-Blame	Self-Distraction	Denial	Substance Use	Avoidance
All (N=398)	Mean	4,32	4,58	4,40	3,89	17,19	5,75	5,80	11,55	4,84	5,32	3,95	14,12	3,14	5,35	4,67	2,97	2,34	18,47
	SD	1,32	1,28	1,32	1,77	3,84	1,35	1,40	2,39	1,42	1,38	1,29	2,97	1,09	1,35	1,33	1,21	0,88	3,72
Harassed (N=127)	Mean	4,36	4,57	4,49	3,80	17,22	5,85	5,98	11,83	4,78	5,46	3,91	14,16	3,20	5,45	4,91	3,12	2,47	19,15
	SD	1,36	1,32	1,39	1,98	4,38	1,29	1,28	2,24	1,52	1,49	1,33	3,29	1,17	1,37	1,42	1,23	1,04	4,10
Not Harassed (N=271)	Mean	4,31	4,58	4,35	3,94	17,18	5,70	5,71	11,41	4,87	5,26	3,97	14,10	3,11	5,30	4,56	2,90	2,28	18,16
	SD	1,30	1,26	1,28	1,66	3,57	1,38	1,45	2,45	1,38	1,32	1,27	2,82	1,05	1,33	1,27	1,19	0,79	3,49
	P-value	*6,93E-01*	*9,07E-01*	*3,44E-01*	*4,82E-01*	*9,24E-01*	*2,94E-01*	*7,14E-02*	*9,77E-02*	*5,51E-01*	*1,63E-01*	*7,01E-01*	*8,48E-01*	*4,06E-01*	*3,13E-01*	*1,66E-02*	*9,87E-02*	*4,17E-02*	*1,31E-02*

Supplementary Table S6. WAI score based on gender.

		WAI 1	WAI 2	WAI 3	WAI 4	WAI 5	WAI 6	WAI 7	WAI Total
**Males**	**Mean**	**7,5**	**8,2**	**5,2**	**5,6**	**3,9**	**6,4**	**3,4**	**40,2**
	SD	1,8	1,5	2,1	0,9	1,4	1,6	0,9	5,8
**Females**	**Mean**	**7,7**	**7,8**	**4,3**	**5,3**	**3,9**	**6,1**	**3,2**	**38,2**
	SD	2,0	2,0	2,2	1,4	1,3	2,0	0,9	7,6
	p-Value	*3,49E-01*	***3,79E-02***	***7,61E-05***	***7,53E-03***	*5,69E-01*	*1,35E-01*	***4,30E-02***	***6,47E-03***

WAI, Work ability Index. WAI1, current work ability compared to the best period of life; WAI2, work ability in relation to job demands; WAI3, the number of current physician-diagnosed illnesses; WAI4, work limitations due to illness; WAI5, sick leave in the past 12 months; WAI6, personal prognosis of work ability over the next two years; and WAI7, mental resources.

Supplementary Table S7. WAI score based on harassment experienced at work (no vs. yes).

		WAI 1	WAI 2	WAI 3	WAI 4	WAI 5	WAI 6	WAI 7	WAI Total
**No**	**Mean**	**7,6**	**8,0**	**5,0**	**5,4**	**3,9**	**6,2**	**3,3**	**39,3**
	SD	2,0	2,0	2,2	1,4	1,4	1,9	0,9	7,5
**Yes**	**Mean**	**7,7**	**7,9**	**3,9**	**5,4**	**3,8**	**6,2**	**3,1**	**38,1**
	SD	1,5	1,3	2,0	1,0	1,2	1,7	0,8	5,2
	p-Value	*7,62E-01*	*9,33E-01*	***9,56E-06***	*7,58E-01*	*3,34E-01*	*6,53E-01*	*8,52E-02*	*9,78E-02*

WAI, Work ability Index. WAI1, current work ability compared to the best period of life; WAI2, work ability in relation to job demands; WAI3, the number of current physician-diagnosed illnesses; WAI4, work limitations due to illness; WAI5, sick leave in the past 12 months; WAI6, personal prognosis of work ability over the next two years; and WAI7, mental resources.

Supplementary Table S8. Spearman's correlation matrices

Not Harassed (N=268)										
**rValue**	**WAI**	**SSS**	**PS**	**PT**	**AV**	**EX**	**AG**	**CO**	**OP**	**NE**
**WAI**	1	-0,043	**0,240**	**0,156**	**-0,174**	**0,265**	**0,165**	0,115	**0,232**	**0,364**
**SSS**		1	**0,256**	**0,318**	**0,356**	0,023	**0,175**	0,001	-0,060	**-0,146**
**PS**			1	**0,403**	0,044	**0,145**	**0,275**	**0,133**	**0,142**	**0,182**
**PT**				1	**0,214**	**0,152**	**0,294**	-0,079	**0,211**	**0,159**
**AV**					1	**-0,166**	-0,029	**-0,249**	**-0,137**	**-0,315**
**EX**						1	**0,424**	**0,208**	**0,244**	**0,206**
**AG**							1	**0,258**	**0,406**	**0,122**
**CO**								1	**0,182**	**0,186**
**OP**									1	**0,221**
**NE**										1
pValue	**WAI**	**SSS**	**PS**	**PT**	**AV**	**EX**	**AG**	**CO**	**OP**	**NE**
**WAI**	1	4,80E-01	**7,11E-05**	**1,05E-02**	**4,22E-03**	**1,11E-05**	**6,62E-03**	5,96E-02	**1,23E-04**	**8,09E-10**
**SSS**		1	**2,15E-05**	**1,06E-07**	**2,05E-09**	7,06E-01	**4,08E-03**	9,82E-01	3,29E-01	**1,71E-02**
**PS**			1	**7,20E-12**	4,77E-01	**1,72E-02**	**4,91E-06**	**2,94E-02**	**2,03E-02**	**2,72E-03**
**PT**				1	**4,31E-04**	**1,30E-02**	**1,00E-06**	1,99E-01	**5,03E-04**	**8,91E-03**
**AV**					1	**6,47E-03**	6,41E-01	**3,82E-05**	**2,48E-02**	**1,45E-07**
**EX**						1	**3,95E-13**	**5,95E-04**	**5,34E-05**	**7,06E-04**
**AG**							1	**1,97E-05**	**4,49E-12**	**4,60E-02**
**CO**								1	**2,76E-03**	**2,24E-03**
**OP**									1	**2,75E-04**
**NE**										1
**Harassed (N=125)**										
**rValue**	**WAI**	**SSS**	**PS**	**PT**	**AV**	**EX**	**AG**	**CO**	**OP**	**NE**
**WAI**	1	-0,019	**0,192**	0,065	**-0,248**	-0,081	-0,018	**0,226**	**0,201**	**0,192**
**SSS**		1	**0,268**	**0,250**	**0,316**	**0,233**	**0,309**	-0,002	0,082	-0,161
**PS**			1	**0,358**	0,082	0,128	**0,181**	**0,260**	**0,205**	**0,279**
**PT**				1	0,117	**0,201**	0,133	-0,009	0,101	0,086
**AV**					1	-0,007	-0,001	**-0,280**	0,091	**-0,450**
**EX**						1	**0,333**	0,054	0,109	**0,176**
**AG**							1	0,161	**0,245**	0,011
**CO**								1	-0,068	**0,423**
**OP**									1	0,001
**NE**										1
pValue	**WAI**	**SSS**	**PS**	**PT**	**AV**	**EX**	**AG**	**CO**	**OP**	**NE**
**WAI**	1	8,31E-01	**3,21E-02**	4,74E-01	**5,23E-03**	3,68E-01	8,41E-01	**1,11E-02**	**2,45E-02**	**3,23E-02**
**SSS**		1	**2,49E-03**	**4,92E-03**	**3,36E-04**	**8,86E-03**	**4,54E-04**	9,79E-01	3,64E-01	7,23E-02
**PS**			1	**4,17E-05**	3,64E-01	1,56E-01	**4,28E-02**	**3,47E-03**	**2,20E-02**	**1,63E-03**
**PT**				1	1,95E-01	**2,45E-02**	1,40E-01	9,20E-01	2,63E-01	3,39E-01
**AV**					1	9,35E-01	9,92E-01	**1,54E-03**	3,14E-01	**1,39E-07**
**EX**						1	**1,46E-04**	5,50E-01	2,25E-01	**4,90E-02**
**AG**							1	7,22E-02	**5,92E-03**	9,03E-01
**CO**								1	4,54E-01	**8,84E-07**
**OP**									1	9,95E-01
**NE**										1

WAI, Work Ability Index (light grey); Brief-COPE (light blue): SSS, Seeking Social Support; PS, Problem-Solving; PT, Positive Thinking; AV, Avoidance; Mini-IPIP (light yellow): EX, Extraversion; AG, Agreeableness; CO, Conscientiousness; OP, Openness; NE, Neuroticism.

Supplementary Table S9. Mantel's correlation strength values

Mantel's	Not Harassed (N=268)		Harassed (N=125)	
	**r**	***p***	**r**	***p***
**WAI**	0,721	*0,001*	0,401	*0,001*
**SSS**	0,428	*0,001*	0,548	*0,001*
**PS**	0,334	*0,001*	0,339	*0,001*
**PT**	0,316	*0,001*	0,409	*0,001*
**AV**	0,418	*0,001*	0,494	*0,001*
**EX**	0,311	*0,001*	0,319	*0,001*
**AG**	0,271	*0,001*	0,355	*0,001*
**CO**	0,280	*0,001*	0,337	*0,001*
**OP**	0,278	*0,001*	0,268	*0,001*
**NE**	0,341	*0,001*	0,361	*0,001*

WAI, Work Ability Index (light grey); Brief-COPE (light blue): SSS, Seeking Social Support; PS, Problem-Solving; PT, Positive Thinking; AV, Avoidance; Mini-IPIP (light yellow): EX, Extraversion; AG, Agreeableness; CO, Conscientiousness; OP, Openness; NE, Neuroticism.
